# Optimizing the ECAP Parameters of Biodegradable Mg-Zn-Zr Alloy Based on Experimental, Mathematical Empirical, and Response Surface Methodology

**DOI:** 10.3390/ma15217719

**Published:** 2022-11-02

**Authors:** Majed O. Alawad, Abdulrahman I. Alateyah, Waleed H. El-Garaihy, Amal BaQais, Sally Elkatatny, Hanan Kouta, Mokhtar Kamel, Samar El-Sanabary

**Affiliations:** 1 Materials Science Research Institute, King Abdulaziz City for Science and Technology (KACST), Riyadh 12354, Saudi Arabia; 2 Department of Mechanical Engineering, College of Engineering, Qassim University, Unaizah 56452, Saudi Arabia; 3 Mechanical Engineering Department, Faculty of Engineering, Suez Canal University, Ismailia 41522, Egypt; 4 Department of Chemistry, College of Science, Princess Nourah Bint Abdulrahman University, Riyadh 11671, Saudi Arabia; 5 Department of Production Engineering and Mechanical Design, Port Said University, Port Fuad 42526, Egypt

**Keywords:** severe plastic deformation, equal channel angular pressing, biodegradable Mg-Zn-Zr alloy, ultrafine-grained structure, corrosion behavior, Response Surface Methodology, genetic algorithm, optimization

## Abstract

Experimental investigations were conducted on Mg-3Zn-0.6Zr alloy under different ECAP conditions of number of passes, die angles, and processing route types, aimed at investigating the impact of the ECAP parameters on the microstructure evolution, corrosion behavior, and mechanical properties to reach optimum performance characteristics. To that end, the response surface methodology (RSM), analysis of variance, second-order regression models, genetic algorithm (GA), and a hybrid RSM-GA were utilized in the experimental study to determine the optimum ECAP processing parameters. All of the anticipated outcomes were within a very small margin of the actual experimental findings, indicating that the regression model was adequate and could be used to predict the optimization of ECAP parameters. According to the results of the experiments, route Bc is the most efficient method for refining grains. The electrochemical impedance spectroscopy results showed that the 4-passes of route Bc via the 120°-die exhibited higher corrosion resistance. Still, the potentiodynamic polarization results showed that the 4-passes of route Bc via the 90°-die demonstrated a better corrosion rate. Furthermore, the highest Vicker’s microhardness, yield strength, and tensile strength were also disclosed by four passes of route Bc, whereas the best ductility at fracture was demonstrated by two passes of route C.

## 1. Introduction

Due to their exceptional properties of high specific strength and low density, which suit the needs of the transportation and lightweight structural industries, magnesium (Mg) alloys have been a source of huge attraction [1,2]. In addition, Mg alloys show a great opportunity in biomedical applications. They look promising to be adopted as bone implant material because of their remarkable advantages compared to traditional biomedical material [3,4,5]. Mg alloys have suitable mechanical properties, good biocompatibility, and extraordinary biodegradable nature [6,7,8]. In addition, Mg alloys are very close in density and elastic modulus to their natural bone counterparts [9]. Moreover, degradation in the body fluid of Mg alloys is one of the most desirable properties of a material used for medical purposes. In addition, Mg alloys’ have high biodegradability in the human body, as they fade after the surgery, which leads to the absence of the need for other surgery to remove the implant [10,11]. Furthermore, Mg is non-toxic and has exceptional biocompatibility; even Mg may possess fortunate impacts on the growth and adhesion of new bone cells [12,13]. 

However, Mg alloys have limitations in clinical applications because of their high corrosion rate in the high chloride physiological systems and rapid degradation rate [14]. Thus, the mechanical integrity damage occurs before the complete curing of bone tissues; Moreover, throughout the corrosion process, a detrimental impact on the Mg alloy as a biodegradable implant is also caused by the development of hydrogen gas bubbles [15,16,17]. Consequently, many approaches from various alloying designs and surface modifications were developed to ameliorate Mg alloys’ corrosion resistance and mechanical properties [15,18,19,20,21,22,23]. Additionally, other attempts were made to adjust the degradation rate by removing impurities and controlling the impurities content ratio [24,25]. Thus, it is crucial for Mg alloys adopted in biomedical applications to possess suitable mechanical properties during their service lifetime, along with being safe bio-alloying elements to guarantee non-toxicity, biocompatibility, and cytocompatibility. Therefore, improving the mechanical and corrosion characteristics of magnesium alloys is of the utmost priority. [19,26,27,28,29]. 

Several studies were performed to improve the mechanical and corrosion properties of Mg alloys, such as effective alloying elements. It was reported that alloying aluminum (Al) resulted in potential toxicity since Al was found to be the reason for Alzheimer’s. Likewise, some rare-earth elements, such as yttrium, were also found to cause liver toxicity [6,30]. Consequently, Al-free Mg alloy is recommended for use with humans [31]. On the other hand, alloying zinc elements (Zn) showed the potential to enhance deformability and D%. In addition, Zn is a vital nutritive element for the body, and its ions can be absorbed effortlessly with no harm done to substantial organs. Likewise, zirconium (Zr) with small contents was reported to be a biocompatible alloying element [5,28,29,32]. Furthermore, Gu et al. discovered that adding Zr and Zn to Mg alloys improved strength, corrosion resistance, and cytocompatibility compared with pure Mg [28,29,32,33].

On the other hand, grain refining is an effective method for improving the mechanical characteristics of Mg alloys [19,28,29,34,35]. It was applied to improve the homogenous nanoscale distributions that claimed uniform corrosion behavior. Therefore, these properties could be attained when using severe plastic deformation (SPD) to reach ultra-fine-grained materials (UFG) [36,37,38,39,40,41,42,43,44,45,46,47]. Additionally, it was stated that the largest positive pitting potentials were associated with the coarse-grained reference alloys and that the ultra-fine-grained Mg alloys induced an enhancement in polarization resistance [48]. Consequently, the Mg-Zn-Zr alloy series (ZKxx) could be a superior alternative in medical implants; it promises a future choice for metallic biodegradable materials [19,28,29,49]. Furthermore, in the modern era of digital databases, generating functional outputs in mathematical forms has become a critical demand [44].

**Figure 1:**
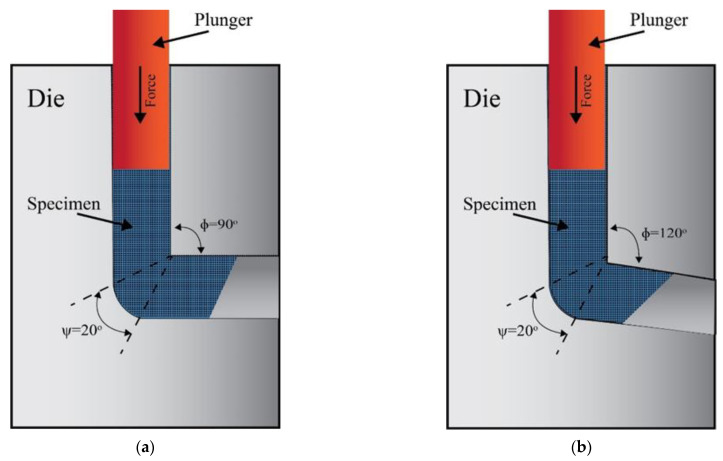
Schematic of the ECAP dies with internal channel angle of (**a**) 90° and (**b**) 120°.

ECAP is the SPD technique that refines metallic materials’ grains the most effective out of all the other SPD approaches and hence improving both the mechanical and electrochemical properties [34,35,50,51,52]. In the ECAP technique, the materials are compelled to exit a die with two parallel channels having an alike cross-sectional profile that intersects at an internal channel angle of ϕ and an angle of curvature of Ψ, as illustrated in Figure 1 [28]. The ECAP route type, in addition to the number of processing passes, has a substantial impact on the mechanical characteristics, crystallographic texture, and microstructural development of the processed billets [53]. The most common ECAP route types are A, Bc, and C [28,53]. In route A, the sample is processed through multiple passes without rotation between the subsequent passes. In contrast, in route Bc, the sample is revolved 90° in the same direction about its longitudinal axis after each pass [28,46,53]. In route C, the sample rotated 180° about the extrusion direction after each pass [53,54]. The imposed equivalent strain ($\varepsilon_{eq}$) can be calculated in terms of the number of ECAP passes (N), die channel angle (ϕ), and the corner curvature angle (Ψ) as shown in Equation (1) [53]. 

A, Bc, and C are the most prevalent ECAP route types [28,53]. Unlike route Bc, which rotates the sample by 90 degrees across its longitudinal axis after each pass, route A processes the sample in successive passes without rotating it [28,46,53]. After each pass along route C, the sample is rotated 180 degrees around the direction of extrusion [53,54]. Equation (1) illustrates how to determine the imposed equivalent strain ($\varepsilon_{eq}$) using the number of ECAP passes (N), die channel angle (ϕ), and corner curvature angle (Ψ) [53].

(1) $$\varepsilon_{eq}=\frac{N}{\sqrt{3}}\left[2\cot\left(\frac{\varphi +\psi }{2}\right)+\psi \;cosec\left(\frac{\varphi +\psi }{2}\right)\right]\;\;\;\;\;\;\;\;\;\;\;$$

On the other hand, the expansion and advancement of useful mathematical insights is a fundamental necessity in the current digital database era [44]. RSM is an effective multivariate statistical method based on an empirical collection of statistical and mathematical instruments that are utilized to create, alter, and finally optimize processes. RSM works by correlating the real and modeled behavior of a response output to several effective input factors based on their own and interaction effects. RSM technique is capable of modeling and optimizing experiments. In addition, GA might be used in optimization to avoid local optimum solutions [55].

Many researchers were optimizing the ECAP conditions using RSM. Daryadel [56] validated the finite element simulation of the ECAP process of AA7075 with copper casing by examining thirty-one tests built by RSM to investigate the ECAP process parameters. The tests were focused on the highest required force and strain, where the main impacts of four chosen significant input factors (friction coefficient, casing thickness, channel, and corner angle) were studied. Consequently, an analysis of variance (ANOVA) of the process variables was conducted to analyze the obtained regression models. Based on the ANOVA analysis, it was assumed that the channel angle affected the response the most; thus, it was the most effective ECAP input parameter. Moreover, the copper casing thickness didn’t show any significant effect on the resultant force response. Likewise, the strain response was affected by channel and corner angle input parameters; conversely, the friction coefficient and copper thickness showed an insignificant effect on the strain response. Finally, the performed optimization reached the optimum predicted ECAP condition aiming at maximizing the forming force and minimizing the strain. The obtained optimum values for channel angle and corner angles were 93.64° and 0°, respectively. Alateyah et al. [57] used RSM, ANOVA, GA, and RSM-GA to optimize the ECAP parameters of pure Mg, and they reported that ECAP processing using a die with ϕ = 90° through 4-passes of route Bc was the most significant parameters in grain reining and Vicker’s microhardness values. Furthermore, ECAP processing using an ECAP die with ϕ = 120° through two passes of route Bc displayed the highest TS, while 4-passes of route C using the 120°-die showed the best D% at fracture. Saleh et al. [58] utilized RSM to optimize the wear resistance of AZ91during ECAP processing using a rotary ECAP die. The RSM findings revealed that the AZ91 wear resistance increased applied load, sliding time, and sliding speed. Furthermore, they reported that the AZ91 wear resistance was improved by increasing the number of processing passes. 

As a result, this study’s objective is to statistically analyze the ECAP performance through tests that were conducted to determine how the ECAP process parameters affected the ZK30 alloy’s mechanical properties and corrosion performance. Experimental investigations were conducted on Zk30 alloy under different ECAP conditions of the ECAP die angles, a number of passes, and processing route types, aiming at reaching the optimum performance characteristics. A complete analysis of the influence of the ECAP conditions on microstructural evolution, mechanical properties, and corrosion performance was presented. The experimental investigation was designed based on RSM that was adopted to classify the optimum ECAP parameters through the analysis of the effect of different ECAP conditions on the numerical responses. Furthermore, analysis of variance (ANOVA) and second-order regression models was obtained to evaluate the optimum ECAP parameters; consequently, GA was used to optimize the ECAP conditions. At last, the optimization of the ECAP responses was enhanced by creating a hybrid RSM-GA, and the subsequent conditions were assessed via GA. 

## 2. Methodology

### 2.1. The Experimental Design Matrix

The most widespread ECAP process parameters reported in previous studies were the number of passes, ECAP die angle, and the type of processing route [53]. The number of passes (one, two, and four passes), ECAP die angle (90° and 120°), and type of processing route (A, Bc, and C) were the levels of the ECAP parameters employed in this investigation, as shown in Table 1.

In this study, RSM was used to provide a design for the combination of the levels of ECAP parameters. Sixteen runs were performed and examined for several ECAP responses, namely, grain size, corrosion response, hardness, and tensile characteristics. Three factors were investigated with a minimum number of experiments using the RSM technique to model a second-order response surface. 

### 2.2. Material and Experimental Procedure

The current study employed a commercial ZK30 alloy (Mg-3Zn-0.6 Zr, wt%). ZK30 billets measuring 20 mm in diameter and 60 mm in length were annealed at 430 °C for 16 h. Two cylindrical channels with an interconnection were used in the ECAP dies, which had internal angles of 90° and 120° and an external die angle of 20°. The ECAP process was applied to the as-annealed (AA) billets under various conditions at a ram speed of 10 mm/min and a temperature of 250 °C. Various routes (A, Bc, and C) were used, as were different passes (one pass (1P), two passes (2P), and four passes (4P)).

The microstructural evolution was studied using a longitudinal cross-section from the center of the ZK30 alloy. The samples were ground incrementally on a grinding wheel spinning at 150 rpm using silicon-carbide sandpaper. Then the samples were polished using diamond suspensions of particle sizes 3 μm, then 1 μm mixed with yellow DP-lubricant. All samples were to have scratch-free surfaces, as seen using a microscope. To that end, a final polishing step was conducted; a 0.05-micron colloidal silica formula was used to provide the final polish. Samples were then etched in a solution of 100 mL ethanol, 5 mL acetic acid (95%), 6 g picric acid, and 10 mL water for 50 s. Finally, to remove the top amorphous layer, the samples were flat ion milled for 30 min using a flat ion milling system. The milling parameters were a grazing angle of 5°, a specimen rotational speed of 0.425 s^−1^, and a beam energy of 2 keV [28,29]. 

The microstructure evolution of the ZK30 biodegradable alloy was investigated using a SU-70 SEM equipped with an EBSD accessory which was used to characterize the microstructural and crystallographic texture evolution as well. The samples investigated by the SEM and EBSD were sectioned from the central longitudinal plane of the ECAPed billets parallel to the pressing direction. The axes of the reference system coincide with the extrusion ECAP direction (ED). The SEM operated at 15 kV and 1.5 nA. The EBSD data were collected in 100 nm increments from the top surface ED plan using HKL Flamenco Channel 5 software (Hitachi, Ltd., Tokyo, Japan) [28,29].

A three-electrode corrosion cell was used to evaluate the corrosion properties of the ECAPed ZK30-Mg alloy. 20 × 30 mm rectangular samples were cleaned with acetone, then rinsed in deionized water after being ground with various silicon-carbide papers up to 4000 grit. A platinum mesh was utilized as a counter electrode; however, the working electrode was an ECAPed ZK30 sample, and the reference electrode was a saturated calomel electrode (SCE). At room temperature, corrosion tests were conducted on ringer lactate corrosive agents. A Luggin capillary was employed to ensure measurement precision and to reduce ohmic drop. An SP-200 Potentiostat was used to record the measurements. Furthermore, a potential scan rate of 0.2 mVs^−1^ using the polarization technique was also used to confirm the steady-state situation. With an open circuit potential and a potential window of ±250 mV, linear potentiodynamic polarization was carried out. At open-circuit potential (E_corr_), electrochemical impedance spectroscopy (EIS) was used with a sinusoidal voltage of ±10 mV and a frequency range of 10 MHz to 100 kHz.

Furthermore, Vicker’s microhardness tests (Hv) were carried out using a digital microhardness tester (Qualitest Canada Ltd, Alberta, Canada) before and after the various ECAP operations, beginning at the sample’s periphery and progressing into the center. A 0.5 kg applied stress was used for 15 s during the microhardness testing. The average outcomes are determined across a minimum of five equispaced indentations. Additionally, utilizing 100 kN universal testing equipment (Instron 4210, Norwood, MA, USA), the room temperature tensile characteristics of ZK30 ECAPed samples were assessed at a strain rate of 10^−3^ s^−1^. The chosen tensile samples were cut to dimensions in accordance with the E8M/ASTM standard and taken from the middle of the ZK30 ECAPed samples. For each processing condition, three tensile samples were examined.

## 3. Response Surface Methodology-Based Experiments

### 3.1. Regression Model

RSM is a very effective tool in most engineering problems that are adopted for model formulation, analysis, design, and enhancement of an optimization process. The interaction between one or more input parameters can also be evaluated using RSM designs. RSM consists of three main steps that were applied in this study. The first step is concerned with the setup of the experimental technique for navigating the process or input factors domain. ECAP independent variables domain was defined as the number of passes, ECAP die angle, and processing route type. The second step focuses on the development of the appropriate model. The models were formed by regression modeling between the input factors and the process responses of grain size, corrosion response, and tensile characteristics. The last step is about getting a three-dimensional surface of the response to fully visualize how the response is behaving as a result of the impact of input variables, which are usually referred to as parameters. Using optimization procedures, the three-dimensional surfaces were used to identify the effect of the ECAP process factors that provide the most suitable output for the ECAP responses [59,60]. 

A regression model provides a function that describes the relationship between a process response (Y) and one or more independent variables, which is obtained by best fitting into first, second, or more-order polynomial equations. Polynomial models of linear, two-way interaction and full quadratic models were adopted in this study to evaluate the obtained experimental data. Equation (2) represents the second-order polynomial mathematical models for the ECAP independent variables and the output response of (Y).

(2) $$Y=\;f\;\left(\mathrm{No}.\;\mathrm{of}\;\mathrm{passes},\mathrm{Die}\;\mathrm{angle},\mathrm{Processing}\;\mathrm{route}\;\mathrm{type}\;\right)+\epsilon$$

where Y is the output response, f represents the ECAP process independent input factors, and ε is the random error distributed about the response Y [61]. 

Analysis of variance (ANOVA) is adopted to investigate the ECAP process independent input factors of the number of passes, ECAP die angle, dummy variable x_1,_ and dummy variable x_2_, and identify which ones of these parameters are significantly impacting the output responses of mechanical properties and corrosion performance [62]. The obtained experimental data were thoroughly studied and analyzed using Stat-Ease Design Expert software (version 13.0.5, Stat-Ease, Inc., Minneapolis, MN, USA). It is a very powerful and efficient computer package used widely in practice for industrial and scientific purposes aiming at designing and optimizing complex systems [63,64]. Design expert provides several types of regression transformation forms, such as linear, square root, natural logarithm, power, and many others. 

The adopted ECAP process input parameters are the number of passes, die angle, and processing route type, as shown in the design of experiments which contains 16 runs (Table 2). Special consideration is placed regarding the processing route type parameter due to the variable’s nature as a categorical type, which is classified into route A, route Bc, and route C. 

One of the adopted techniques to transform a categorical variable into a numerical one is dummy coding. It is based on a binary coding system as it provides all of the crucial information about group membership using only zeros and ones. It is one of the adopted methods for employing variables of categorical predictor nature in various types of prediction models of linear and other regressions. Dummy variables are used to denote a category variable that was transformed through dummy coding. To build dummy variables that are exhaustive and mutually exclusive and relate to a specific category variable with K classifications, a series of K-1 dummy variables is required [65]. As illustrated in the matrix in Equation (3), the dummy variables for the category variables route A, Bc, and C were coded as x_1_ and x_2_.

(3) $$\begin{array}{cc} x_{1} & x_{2} \end{array}\;\begin{array}{c} A\\ \mathrm{Bc}\\ C \end{array}=\left[\begin{array}{cc} 1 & 0\\ 0 & 1\\ 0 & 0 \end{array}\right]$$

where route type A has x_1_ = 1 and x_2_ = 0, if route type Bc then x_1_ = 0 and x_2_ = 1, and if route type C then x_1_ = 0 and x_2_ = 0.

The finest regression models that could be statistically significant were found after numerous iterations of regression transformation forms and research into interactions between independent variables of ECAP parameters. 

### 3.2. Genetic Algorithm

A genetic algorithm (GA) is a widely used method in various engineering and science-based applications. GA is characterized by its smart, effective, and inexpensive way of tackling real-life optimization problems. GA provides optimum algorithms along with a random number of generations in each individual. On the other hand, a common convention algorithm adopts a predetermined strategy for establishing the following generation and only generates a single point. Every generation evaluates an individual’s fitness functions. The convergence of the results is ensured by GA, which adopts certain criteria to arrive at a value aiming at a global minimum for a fitness function [60,66]. 

## 4. Results and Discussion

The experimental findings [28,29] of the different studied ECAP process factors were used for the development of a qualitative and quantitative assessment strategy to examine how the ECAP parameters affected the ZK30 alloy regarding the evolution of grain size, electrochemical response, and mechanical characteristics of the ZK30 alloy. Several trials of regression transformation form and interactions of independent variables were tested thoroughly for modeling the output responses of the ECAP. The generated models in the experimental investigation were proven to show statistical significance. 

### 4.1. Experimental Results and RSM

#### 4.1.1. Microstructural Evolution

**Figure 2:**
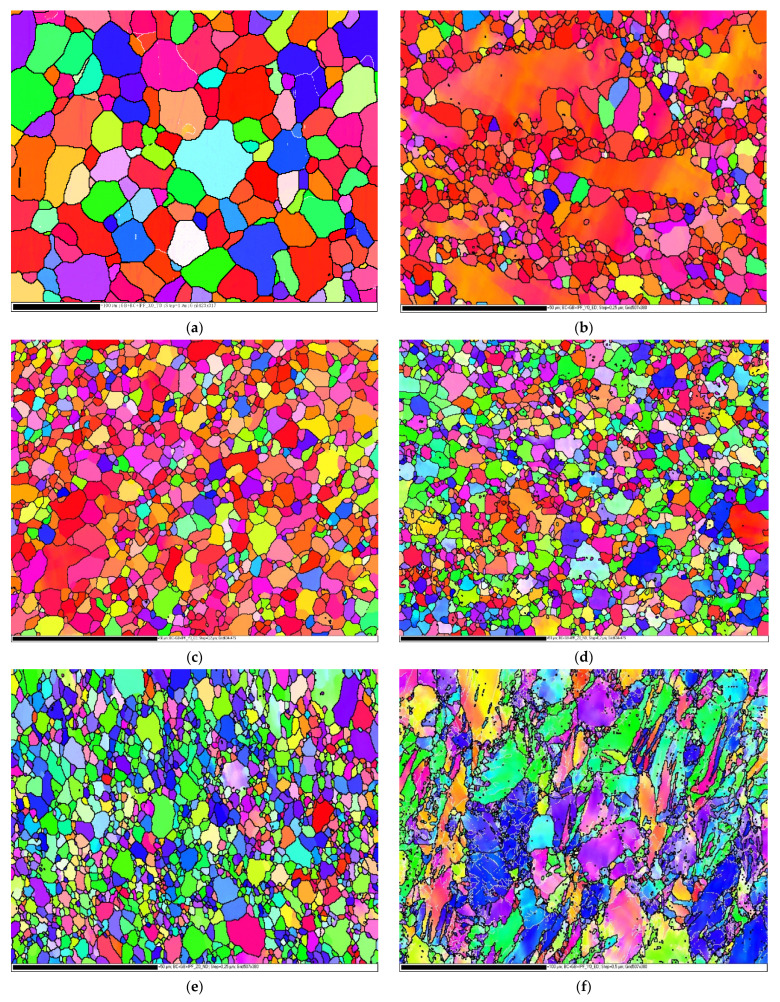

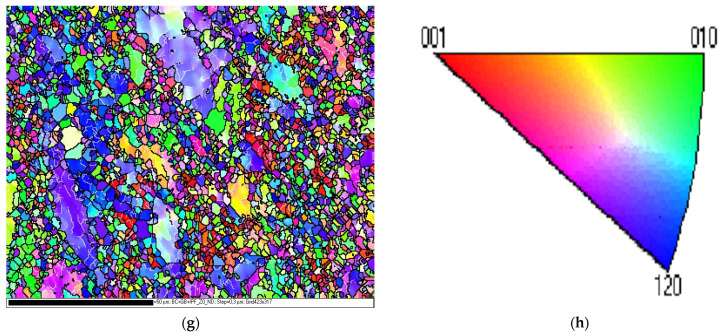
EBSD orientation maps for the AA- ZK30 (**a**) and after the ECAP processing through 1-P (**b**), 4-A (**c**), 4-Bc (**d**), 4-C (**e**) using the 90°-die and 1-P (**f**), 4-Bc (**g**) using the 120°-die and the inverse pole figure (IPF) coloring triangle is shown in (**h**), red: [001]; blue: [120]; and green: [010].

EBSD was adopted to assess the grain structure of the ZK30. Figure 2 shows the orientation maps of AA-ZK30 after different ECAP processes, all relative to the ED [28,29]. Based on the results of the experiments, Table 3 depicts the average grain size of ZK30 alloy billets processed by AA and ECAP. As seen in Figure 2a, the orientation map of AA-ZK30 revealed equiaxed coarse grains and certain regions with fine grains. Grain refinement can be observed after the first ECAP pass, utilizing die angles of 90° and 120°, as revealed in Figure 2b,f, respectively. However, the die angle of 120° produces a coarser grain size compared with 90° as the latter experienced a higher strain relative to the first. Furthermore, augmenting the number of passes to four using different routes of A (Figure 2c), Bc (Figure 2d), and C (Figure 2f) resulted in further refinement. However, route Bc showed finer and more homogeneous grain distribution, as shown in Figure 2d. Furthermore, by inspection of Figure 2d, it is clear that 4Bc processing using the 90°-die leads to increasing areas of ultrafine grain structure (UFG) despite the existence of minor areas of coarser grains. Accordingly, the existing coarse grains of the 4Bc condition resulted in increasing the average grain size up to 1.94 µm as displayed in Table 3. In addition, it is clear from Figure 2 and Table 3 that in terms of grain refinement, route Bc is the most efficient. On the other hand, processing through multiple passes indicated that the die angle had an insignificant effect on the average grain size since processing through 4-Bc with the 90°-die and 120°-die reduced grain size by 92.7% and 92.8%, respectively, as relative to the AA equivalent.

The predicted inverse model of ZK30 grains size performed from the ECAP process is presented in Equation (4), whereas the ANOVA results are shown in Table A1 (Appendix A).

1/Grain Size = 0.617014 − 0.0643169 × No. of Passes − 0.00418436 × Die Angle + 0.0693044 × x_1_ − 0.0448229 × x_2_ + 0.00129203 × No. of Passes × Die Angle − 0.0415678 × No. of Passes × x_1_+ 0.0250216 × No. of Passes × x_2_ (4)

Referring to Table A1, the coefficient of determination (R^2^) of grain size is 0.9857, and the adjusted R^2^ is 0.9732, which is close to and within 0.2 of the predicted R^2^ value of 0.9155. Therefore, the obtained high values of R^2^, adjusted R^2^, and predicted R^2^ for grain size indicate that the created model is desirable. The model terms A, B, C, AB, AC, and AD all have *p*-values that are lower than 0.05, implying that they are significant. 

Similarly, the grain size model is significant with *p*-values less than 0.05, which designates that altering an input ECAP parameter would significantly affect the grain size [67], indicating that this model is satisfactory at a 95% confidence level [68]. The number of passes of the ECAP process has the greatest impact on grain size, followed by ECAP die angle, and finally, dummy variables x_1_ and x_2_. The adequate precision is 29.85, which is greater than four, implying that there is an adequate signal and the model could be used for navigating the design space [69].

**Figure 3:**
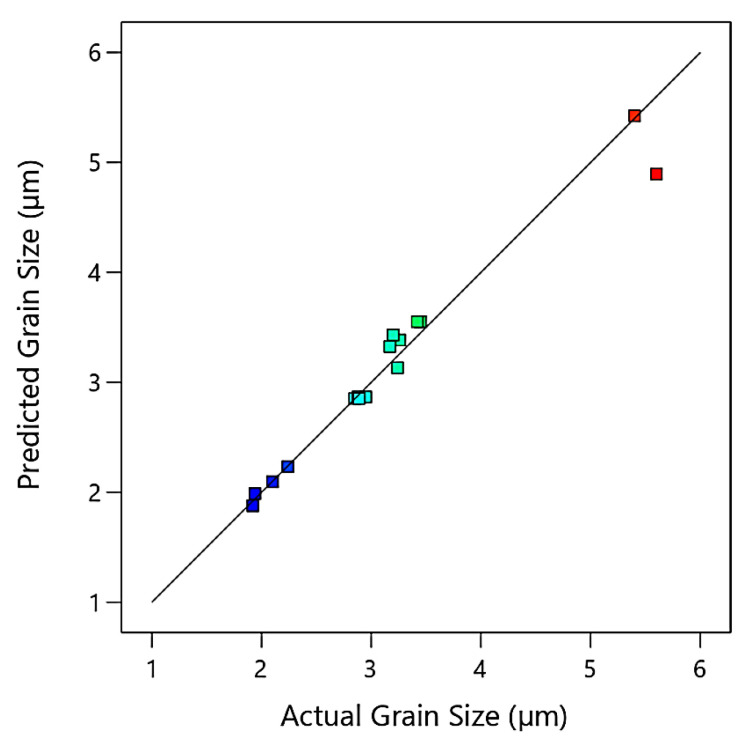
Predicted versus actual values of grain size, where the blue points are for minimum output value and gradually changed to red points for maximum output value.

Figure 3 is a comparison between actual experimental data and predicted values of grain size for ZK30 samples calculated by the regression model for a course of 16 iterations. It could be deduced from the figure that the bulk of the anticipated findings matches the actual experimental data extremely well with a narrow slight deviation. In addition, it indicates that the obtained regression model is adequate and could be useful for predicting the optimization of ECAP parameters for the best grain size. 

**Figure 4:**
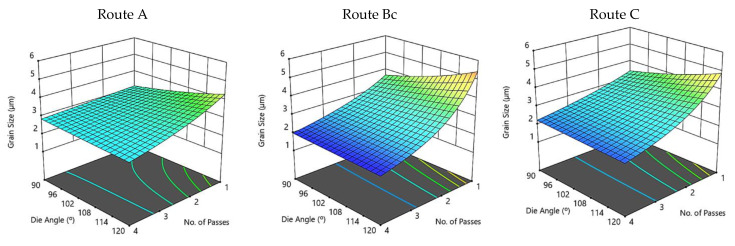
Three-dimensional plot of grain size with ECAP die angle and number of passes at routes A, Bc, and C.

Figure 4 illustrates three-dimensional interaction viewgraphs on the impact of the ECAP parameters on the output response of average grain size. It shows response surface plots for interaction viewgraphs between two variables, ECAP die angle and number of ECAP passes, while fixing the processing route variable. For route A, the increase of ECAP die angle resulted in increasing the grain size. Changing the number of passes has affected the grain size minimally. The minimum optimum grain size is 2.89 µm at route A, which is obtained at four passes and a 90° ECAP die angle. The die angle of the ECAP process at route Bc is proportional to grain size; moreover, the ECAP grain size shrank as the number of passes increased. In this context, the minimum optimum grain size is 1.92 µm at route Bc, which is obtained at four passes and 120° ECAP die angle. Likewise, the effect of die angle and number of passes at route C on grain size is similar to those obtained by route Bc. The minimum optimum grain size is 2.1 µm at route C, which is obtained at four passes and 120° ECAP die angle. 

#### 4.1.2. Corrosion Behavior 

The corrosion response of ZK30 was explained by corrosion rate and corrosion resistance. The predicted inverse model of corrosion rate is presented in Equation (5) and the linear quadratic model of corrosion resistance is presented in Equation (6).

1/Corrosion Rate = 16.1243 − 8.08576 × No. of Passes − 0.0752773 × Die Angle + 0.145218 × x_1_ − 40.4689 × x_2_ + 0.0303579 × No. of Passes × Die Angle + 16.7947 × No. of Passes × x_2_ + 0.333308 × Die Angle × x_2_ +0.932053 × No. of Passes^2^ − 0.134907 × No. of Passes × Die Angle × x_2_ (5)

Corrosion Resistance = 863.142 − 869.61 × No. of Passes + 10.6176 × Die Angle − 24.5789 × x_1_ − 2116.51 × x_2_ − 3.14036 × No. of Passes × Die Angle + 612.013 × No. of Passes × x_2_ + 18.0897 × Die Angle × x_2_ + 210.811 × No. of Passes^2^ − 5.19565 × No. of Passes × Die Angle × x_2_ (6)

Table A2 (Appendix A) presents the analysis of variance (ANOVA) results for ZK30 corrosion characteristics after ECAP processing. The individual model coefficients, interaction, and quadratic terms, as well as the appropriate *p*-value from the ANOVA for corrosion rate and resistance, are shown in Table A2. In the case of corrosion rate, the model terms A, D, AB, AD, A^2^, and ABD all have *p*-values that are lower than 0.05, implying that they are significant. In the case of pitting corrosion resistance, the model terms A, B, AB, and A^2^ all have *p*-values that are lower than 0.05, implying that they are significant. Similarly, both corrosion rate and resistance models are significant with *p*-values less than 0.05, which designates that altering an input ECAP parameter significantly affects the corrosion rate and corrosion resistance quality criteria [67], indicating that these models are satisfactory at a 95% confidence level [68]. The number of passes of the ECAP process, factor A, has the greatest impact on corrosion rate and corrosion resistance. The adequate precision is 34.17 and 11.85 for corrosion rate and corrosion resistance, respectively, which is greater than four, implying that there is an adequate signal and the model could be used for navigating the design space [69]. The coefficient of determination (R^2^) values is 0.991 and 0.9456 for corrosion rate and resistance, respectively. Additionally, the adjusted R^2^ of the corrosion rate is 0.9775, which is close to and within 0.2 of the predicted R^2^ value of 0.9846. In addition, the adjusted R^2^ of corrosion resistance is 0.864, which is close to and within 0.2 of the predicted R^2^ value of 0.8798. Therefore, the obtained high values of R^2^, adjusted R^2^, and predicted R^2^ for corrosion rate and resistance indicate that the created model is desirable. The corrosion rate’s lack of fit *p*-value is 0.6, which is more than 0.05, indicating an insignificant lack of fit and a good model [67].

**Figure 5:**
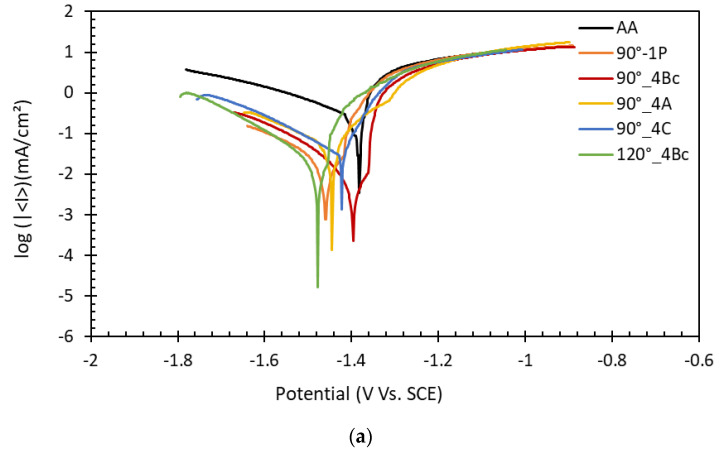

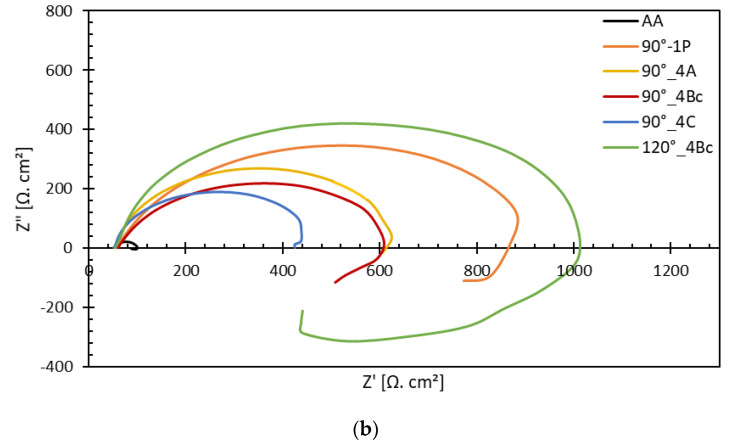
Corrosion measurements (**a**) potentiodynamic polarization curves, and (**b**) Nyquist plot of AA and ECAPed billets processed via various ECAP conditions.

Electrochemical experiments were carried out on the biodegradable ZK30 ECAPed at various process settings, as well as on the AA, as found in [28,29]. The measurements were carried out using ringer lactate electrolytic solution, as it mimics the human body fluids. Figure 5 illustrates the potentiodynamic polarization curves (a) and Nyquist plots (b) of ZK30 for the different ECAP process parameters. 

The Tafel plot is a reliable method of corrosion resistance investigation [52]. As illustrated in Figure 5a, the 1P using the 90°-die condition showed a significant reduction in corrosion potential relative to the AA counterpart, along with a notable noble corrosion current shift toward the lower current density (I_corr_). Additional ECAP processing passes, 4P using the 90°-die, through different routes resulted in an additional drop of corrosion current compared to the 1P, except for the 90°_4C. In addition, increasing the die angle up to 120° using route Bc resulted in significant corrosion I_corr_ reduction compared to 90°_4Bc. The I_corr_ reduction could be considered a dependable indicator for decreasing the corrosion rate. However, increasing the die angle to 120° (120°_4Bc) resulted in shifting the corrosion potential E_corr_ to more negative values. 

The EIS results, Nyquist plots, were similar; however, the semicircle diameters of were dissimilar. The semicircle diameter is significantly connected to the charge resistance and, consequently, the corrosion rate. Consequently, the largest semicircle diameter represents the best corrosion resistance [70]. As shown in Figure 5b, the response of the AA- ZK30 billets was the smallest semicircle compared to the ECAPed billets. Moreover, the ECAP processing through 1P using the 90°-die showed a substantial rise in the semicircle diameter. However, further ECAP processing passes, four passes, caused an increase in the semicircle diameter compared to 1P, which might be attributable to strain buildup, which increases dislocation density [28,70,71]. On the other hand, the different routes have a substantial impact on the corrosion resistance, as shown in Figure 5b. Using route C resulted in the smallest semicircle diameter; however, route A shows an insignificant increase in the semicircle diameter compared to the 4Bc counterpart. In addition, processing through the ECAP die with 120°, 4Bc increases the semicircle diameter compared to the sample processed through 4Bc using the 90°-die which indicated higher corrosion resistance. The 120°-die’s improved corrosion resistance compared to the 90°-die can be ascribed to an improvement in dislocation density during ECAP processing via the 90°-die because of the increased plastic strain, as reported earlier in the literature [34,72]. Accordingly, increasing the dislocation density resulted in decreasing the corrosion resistance. 

**Figure 6:**
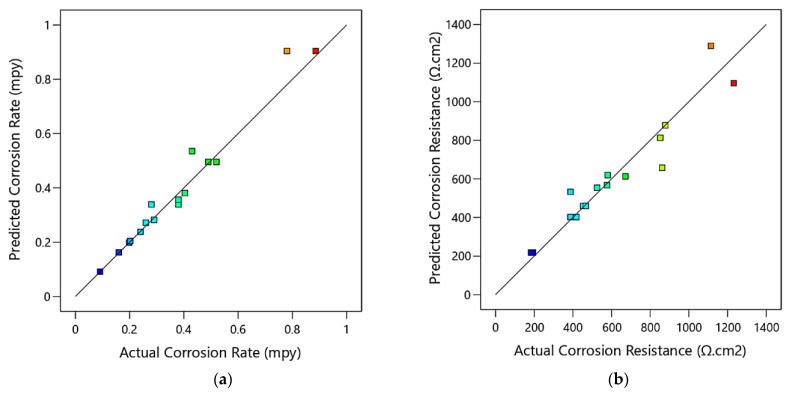
Predicted versus actual values of the ECAP corrosion rate (**a**) and corrosion resistance (**b**), where the blue points are for minimum output value and gradually changed to red points for maximum output value.

**Figure 7:**
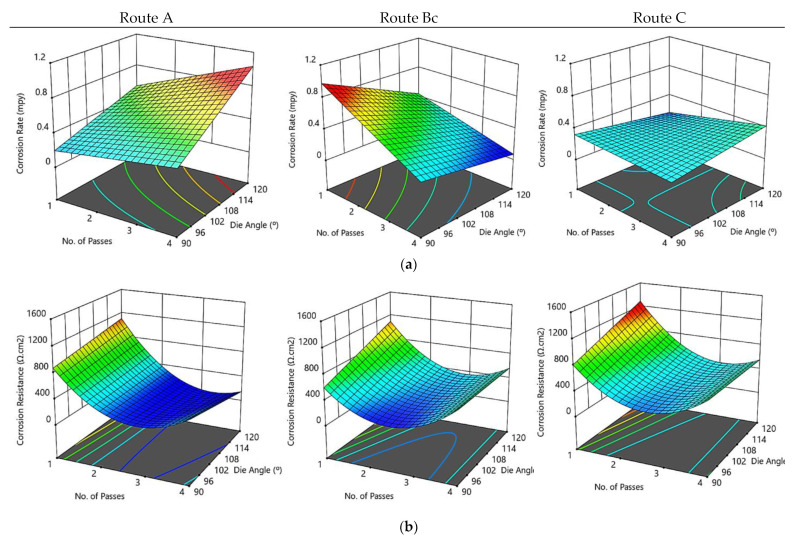
Three-dimensional plot of corrosion rate (**a**) and corrosion resistance (**b**) with ECAP die angle and number of passes at routes A, Bc, and C.

Figure 6 is a comparison between actual experimental data and predicted values of corrosion rate and corrosion resistance of the ZK30 samples calculated by the regression model, for a course of 16 iterations. It could be deduced from the figure that the bulk of the anticipated findings matches the actual experimental data extremely well. In addition, it indicates that the obtained regression model is adequate and could be useful to predict the optimization of ECAP parameters for the best grain size. Figure 7 illustrates three-dimensional interaction viewgraphs on the effect of the ECAP parameters on the output response of corrosion rate and resistance. It shows response surface plots for interaction viewgraphs between two variables, ECAP die angle and number of ECAP passes, while fixing the processing route variable. 

For route A, the increase of the ECAP die angle resulted in increasing the corrosion rate. The change of the number of passes has affected the corrosion rate minimally. The minimum optimum corrosion rate is 0.198 mils per year (mpy) at route A, which is attained at one pass and a 90° ECAP die angle. The number of passes of the ECAP process at route Bc is inversely proportional to corrosion rate; moreover, the ECAP grain size decreased as the number of passes increased. Additionally, the ECAP die angle affects slightly the corrosion rate. In this context, the minimum optimum corrosion rate is 0.091 mpy at route Bc, which is obtained at four passes and 90° ECAP die angle (Figure 7a). Similarly, there is a minor effect at route C of die angle and number of passes on corrosion rate. The aforementioned results suggest that the improved corrosion rate following ECAP processing might be attributable to the obtained fine grain size (Figure 2), which is consistent with the potentiodynamic polarization findings.

Regarding corrosion resistance (Figure 7b), the corrosion resistance decreased as the ECAP number of passes at route A increased. Altering the die angle affects the corrosion resistance minimally. The maximum optimum corrosion resistance is 878 Ω·cm^2^ at route A, which is attained at one pass and a 90° ECAP die angle. The corrosion resistance showed a decline with augmenting the number of passes nearly up to two passes, then it improved with augmenting the number of ECAP passes at route Bc. The ECAP die angle has a minor effect on corrosion resistance. In this context, the maximum optimum corrosion resistance 1232 Ω·cm^2^ at route Bc, which is attained at one pass and 120° ECAP die angle. Similarly, the effect of die angle and number of passes at route C on corrosion resistance is similar to those obtained by route Bc. The maximum optimum corrosion resistance is 1114 Ω·cm^2^ at route C, which is attained at one pass and 120° ECAP die angle. 

#### 4.1.3. Mechanical Properties

##### Hardness Distribution

The inverse square root predicted models of hardness response at the center and edge of the ECAP specimen of ZK30 are presented in Equations (7) and (8).

1/Sqrt (Hardness at center) = +0.125954 − 0.009301 × No. of Passes − 5.33485 × 10^−6^ × Die Angle −0.021424 × x_1_ − 0.001141 × x_2_ +0.000240 × Die Angle × x_1_ +0.001192 × No. of Passes^2^ (7)

1/Sqrt (Hardness at Edge) = +0.101197 + 0.002123 × No. of Passes + 0.000094 × Die Angle − 0.016928 × x_1_ + 0.000252 × x_2_ −0.000035 × No. of Passes × Die Angle − 0.000680 × No. of Passes × x_2_ + 0.000180 × Die Angle × x_1_ (8)

The analysis of variance (ANOVA) results for ZK30 of the ECAP parameters on hardness response is represented in Table A3. The individual model coefficients of hardness response, interaction, and quadratic terms, as well as the appropriate *p*-value from the ANOVA for hardness at the center and edge, are shown in Table A3 (Appendix A).

In the instance of hardness at the edge, the *p*-value of every model term is lower than 0.05, indicating that every model term is significant. In contrast, the hardness at the center case has model terms for A, B, C, BC, and A^2^ that are smaller than 0.05, suggesting that these model terms are significant. Similarly, both hardness at the center and edge models are significant with *p*-values less than 0.05, which designates that altering an ECAP parameter significantly affects both the hardness at the center and edge quality criteria [67], indicating that these models are satisfactory at a 95% confidence level [68]. The number of passes of the ECAP process has the greatest impact on both the hardness at the center and edge, followed by the ECAP die angle. The adequate precision values are 24.5 and 26.68 for the hardness at the center and edge, respectively, which is more than four, implying that there is an adequate signal and the model could be used for navigating the design space [69]. The coefficient of determination (R^2^) values is 0.984 and 0.9825 for the hardness at the center and edge, respectively. Additionally, the adjusted R^2^ of the hardness at the center is 0.9743, which is close to and within 0.2 of the predicted R^2^ value of 0.9481. In addition, the adjusted R^2^ of the hardness at the edge is 0.9671, which is close to and within 0.2 of the predicted R^2^ value of 0.9245. Therefore, the obtained high values of R^2^, adjusted R^2^, and predicted R^2^ for both the hardness at the center and edge indicate that the created model is desirable.

**Figure 8:**
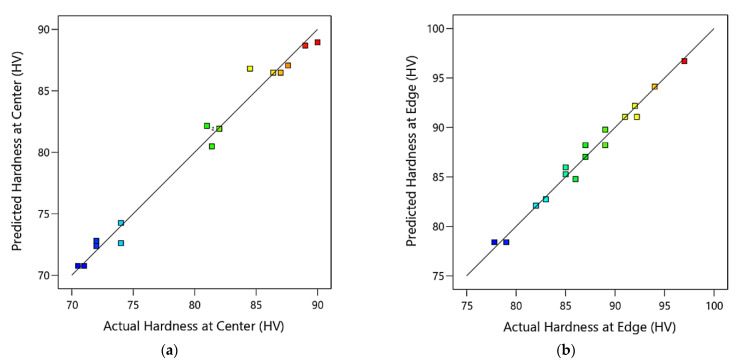
Predicted versus actual values of the ECAP hardness at the center (**a**), and the edge (**b**), where the blue points are for minimum output value and gradually changed to red points for maximum output value.

The relationships between the actual experimental data and the predicted response values calculated by the regression model of the hardness of the ZK30 at the center and edge are shown in Figure 8 for a course of 16 iterations. It could be deduced from the figure that the bulk of the anticipated findings matches the actual experimental data extremely well. Additionally, it indicates that the obtained regression models are adequate and could be useful to predict the optimization of ECAP parameters for the best hardness at the center and edge.

**Figure 9:**
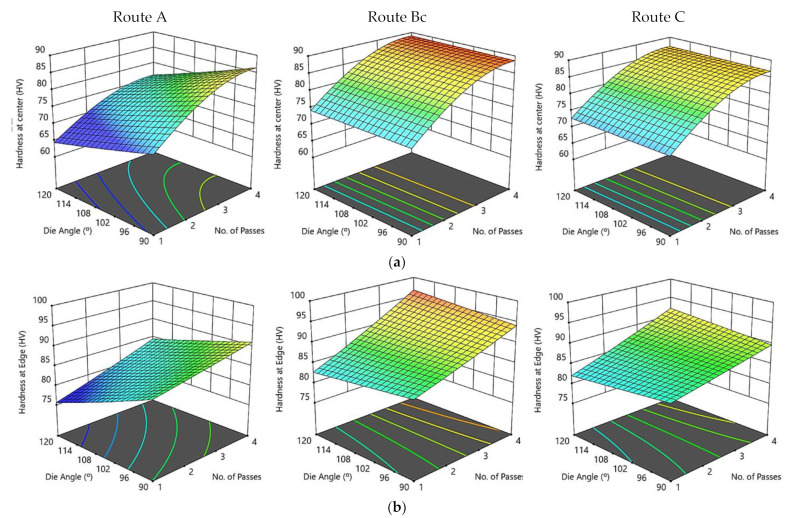
A three-dimensional plot of hardness at the center (**a**) and edge (**b**) with the ECAP die angle and number of passes for routes A, Bc, and C.

Figure 9 illustrates three-dimensional interaction viewgraphs on the effect of the ECAP parameters on the output response of hardness at the center and edge. It shows response surface plots for interaction viewgraphs between two variables, ECAP die angle and number of ECAP passes, while fixing the processing route variable. It can be seen that there is a minor effect of the ECAP die angle on hardness at the center of the specimen at routes A, Bc, and C. However, the hardness at the center showed an increase when the ECAP number of passes increased. The maximum hardness at the center at route A is attained at 87 HV with four passes and a 90° die angle. Likewise, the maximum hardness at the center at route Bc is attained at 90 HV with four passes and a 120° die angle. Regarding route C, the maximum hardness at the center is attained at 87.6 HV with four passes and 120° die angle (Figure 9a).

Regarding route A, the increase in the ECAP number of passes resulted in increasing the hardness at the edge; on the other hand, the hardness at the edge decreased with the increase in the ECAP die angle. The maximum optimum hardness at the edge at route A is attained at 92.2 HV with 4-passes, and 90° ECAP die angle. Regarding routes Bc and C, it is noticed that there is a minor effect of the ECAP die angle on hardness at the edge; in addition, a proportional effect between hardness at the edge and the number of passes is observed (Figure 9b). As a result, the maximum optimum value of hardness at the edge for routes Bc and C is 97 HV, and 92 HV, respectively, which is obtained at four passes and 120° ECAP die angle.

From the experimental and aforementioned findings, the central areas clearly exhibited lower hardness values relative to the areas at the peripheries, which could be attributed to the friction between the internal die walls and the ZK30 billets. This finding agrees with a previous study in literature [35]; in addition, increasing the number of ECAP passes resulted in increasing the hardness distribution homogeneity at both peripheral and central areas. Moreover, increasing the die angle up to 120° in both regions resulted in decreasing the hardness, which could be attributed to the decrease in the plastic strain [35]. Furthermore, increasing the number of passes resulted in hardness improvement, which could be argued to be the result of strain hardening [57,73]. 

##### Tensile Properties

The calculated tensile responses of the ZK30 specimen are yield strength (YS), tensile strength (TS), and ductility percentage (D%). Equations (9)–(11) represent the three models of tensile responses.

YS = 134.224 − 13.603 × No. of Passes − 0.402982 × Die Angle − 1.34108 × x_1_+ 3.98405 × x_2_ + 0.0973018 × No. of Passes × Die Angle + 0.939662 × No. of Passes^2^ (9)

TS = 446.709 − 25.5164 × No. of Passes − 1.26603 × Die Angle − 9.29292 × x_1_ − 4.91157 × x_2_ + 0.293265 × No. of Passes × Die Angle + 2.6981 × No. of Passes × x_2_ (10)

D% = 25.4808 + 2.07884 × No. of Passes + 0.082289 × Die Angle − 3.08458 × x_1_+ 0.894089 × x_2_ + 1.49867 × No. of Passes × x_1_ − 1.51498 × No. of Passes × x_2_ − 0.623325 × No. of Passes^2^ (11)

The analysis of variance (ANOVA) results for ZK30 of the ECAP parameters on characteristic tensile responses are represented in Table A4 and Table A5 (Appendix A). The individual model coefficients of characteristic tensile responses, interactions, and quadratic terms, as well as the appropriate *p*-value from the ANOVA for YS, TS, and D%, are shown in Table A4 and Table A5. In the case of YS, the *p*-values of A, B, D, and AB are less than 0.05, indicating that these model terms are significant. In the case of TS, the *p*-values of A, B, C, and AB are less than 0.05, indicating that these model terms are significant. In the case of the D% percentage, the *p*-values of A, B, D, AC, AD, and A^2^ are less than 0.05, indicating that these model terms are significant. However, the other model terms with *p*-values greater than 0.05 are insignificant. Similarly, the YS, TS, and D% percentage models are significant with *p*-values less than 0.05, which designates that changing an input ECAP parameter has a significant impact on the YS, TS, and D% quality criteria [67], indicating that these models are satisfactory at a 95% confidence level [68]. The ECAP die angle has the greatest impact on YS, TS, and D percentages followed by the number of ECAP passes. 

The adequate precision values are 12.5, 19, and 29.4 for YS, TS, and D%, respectively, which is greater than four, implying that there is an adequate signal and the model could be used for navigating the design space [69]. The coefficient of determination (R^2^) values is 0.9321, 0.97, and 0.9848 for the YS, TS, and D%, respectively. Additionally, the adjusted R^2^ of YS is 0.886, which is close to and within 0.2 of the predicted R^2^ value of 0.742. In addition, the adjusted R^2^ of TS is 0.95, which is close to and within 0.2 of the predicted R^2^ value of 0.906. Moreover, the adjusted R^2^ of D% is 0.97, which is close to and within 0.2 of the predicted R^2^ value of 0.94. Therefore, the obtained high values of R^2^, adjusted R^2^, and predicted R^2^ for the YS, TS, and D% indicate that the created model is desirable. The YS, TS, and D% lack of fit *p*-values is greater than 0.05, indicating an insignificant lack of fit and a good model [67].

**Figure 10:**
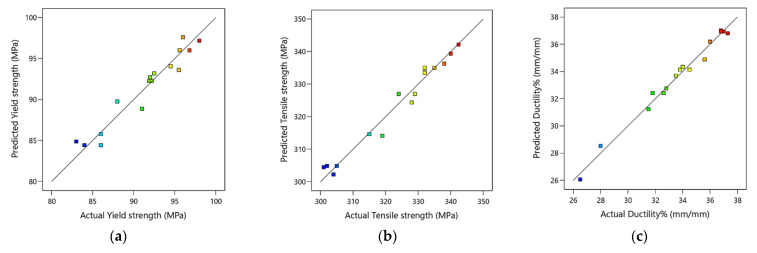
Predicted versus actual values of the ECAP YS (**a**), TS (**b**), and D% (**c**), where the blue points are for minimum output value and gradually changed to red points for maximum output value.

The relationships between the actual experimental data and the predicted response values calculated by the regression model of the ZK30′s YS, TS, and D% are shown in Figure 10 for a course of 16 iterations. It could be deduced from the figure that the bulk of the anticipated findings matches the actual experimental data extremely well., especially in the case of YS and TS. Additionally, it indicates that the obtained regression models are adequate and could be useful for predicting the optimization of ECAP parameters for the best YS, TS, and D percentages. 

**Figure 11:**
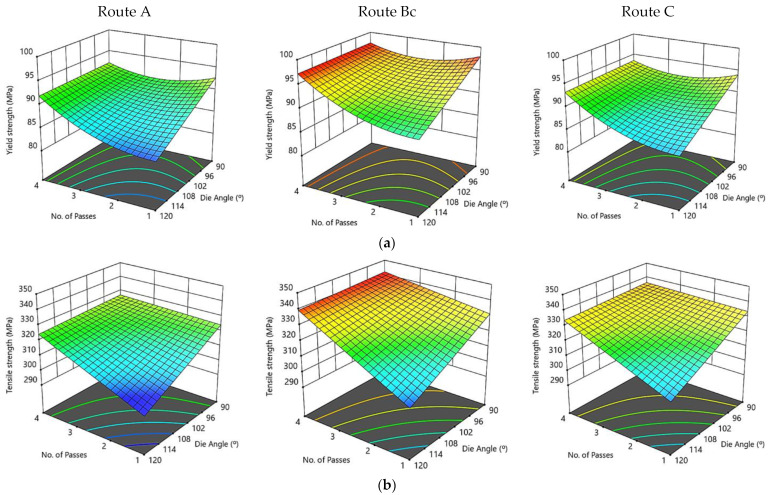

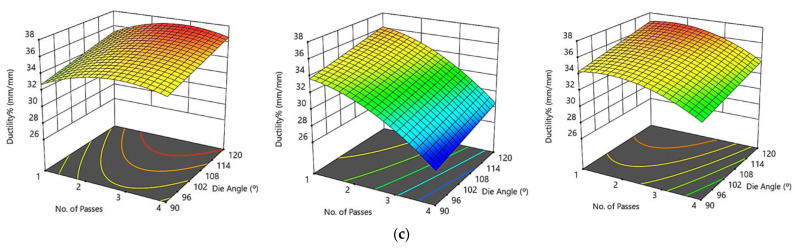
Three-dimensional plot of YS (**a**), TS, (**b**) and D% (**c**) with the ECAP die angle and number of passes at routes A, Bc, and C.

Figure 11 illustrates three-dimensional interaction viewgraphs on the effect of the ECAP parameters on the output response of YS, TS, and D%. It shows response surface plots for interaction viewgraphs between two variables, ECAP die angle and number of ECAP passes, while fixing the processing route variable. It is observed that the ECAP die angle and number of passes showed a similar effect on YS at different routes. It is seen that for routes A, Bc, and C, the YS increases with the decrease in the ECAP die angle and the increase in the number of passes. The maximum YS at route A is attained at 92.2 MPa with four passes and a 90° die angle. Moreover, the maximum YS at route Bc is attained at 98 MPa with four passes and a 120° die angle. Regarding route C, the maximum YS is attained at 95.5 MPa with four passes and a 90° die angle (Figure 11a). The TS of the ECAP process showed an alike trend for routes A, Bc, and C. The values of the maximum TS are 329 MPa, 342.4 MPa, and 338 MPa for routes A, Bc, and C, respectively, which were attained with four passes and 90° ECAP die angle (Figure 11b). For routes A, Bc and C, the D% decreased with the decrease of the ECAP die angle. The maximum D% at routes Bc and C is attained at 36.19 and 36.81, respectively, with one pass and 120° die angle. Moreover, the maximum D% at route A is attained at 34.79 with two passes and a 120° die angle (Figure 11c). 

From the tensile results, it can be observed that ECAPed ZK30 billets displayed a momentous improvement in YS and TS without showing a substantial drop in D% in comparison with the AA counterparts. Furthermore, it was shown that the processing conditions at 90° ECAP die angle, four passes, and route Bc revealed the best YS. Consequently, route Bc showed to be the furthermost efficient route in enhancing the YS because of the substantial grain size decrease, as shown in Figure 2. The UFG obtained through ECAP processing via multiple passes could be associated to be the main reason for hindering the dislocation motion [74,75]. Therefore, the grain refining mechanism is the most efficient strengthening mechanism, which led to an enhancement in the mechanical properties. Additionally, the adoption of the 90° die angle caused a significant improvement in the YS_,_ which could be attributed to the higher plastic strain. Furthermore, the shear strain accumulation resulting from the ECAP passes of up to four passes could be assigned to the dislocation density growth, which hinders the dislocation mobility [76]. Moreover, the D% reduction after the ECAP processing could be associated with grain refinement. Additionally, better D% was observed at route Bc with a 120° die angle and four passes, compared with route Bc with a 90° die angle and four passes, which could be assigned to imposing lower strain as reported in [29]. In the same context, route Bc exhibited the highest grain refinement; therefore, it shows lower D% compared to the remaining studied route types of A and C. Consequently, route Bc could be considered the most effective route type in this perspective.

### 4.2. Genetic Algorithm Results

#### 4.2.1. Optimization of Grain Size

A minimization was considered for the grain size response presented in Equation (4), which was set to be the objective function using GA and subjected to the ECAP boundary conditions of number of passes, ECAP die angle, dummy variable x_1_, and dummy variable x_2_. It is presented as follows:

Minimize grain size (number of passes. ECAP die angle, x_1_, x_2_)

Subjected to ranges of ECAP conditions:

1 ≤ No. of passes ≤ 4 (pass);

90 ≤ die angle ≤ 120 (°);

Dummy variable x_1_ ϵ [0, 1];

Dummy variable x_2_ ϵ [0, 1].

**Figure 12:**
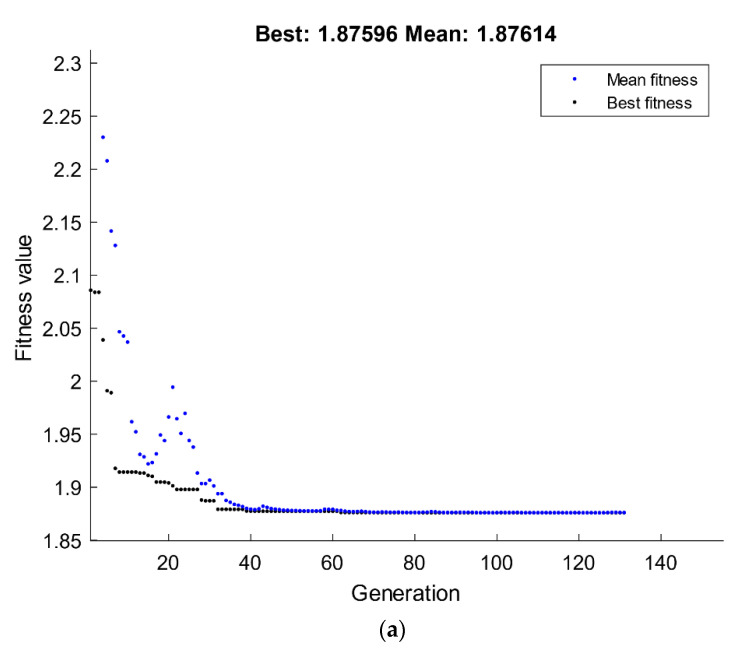

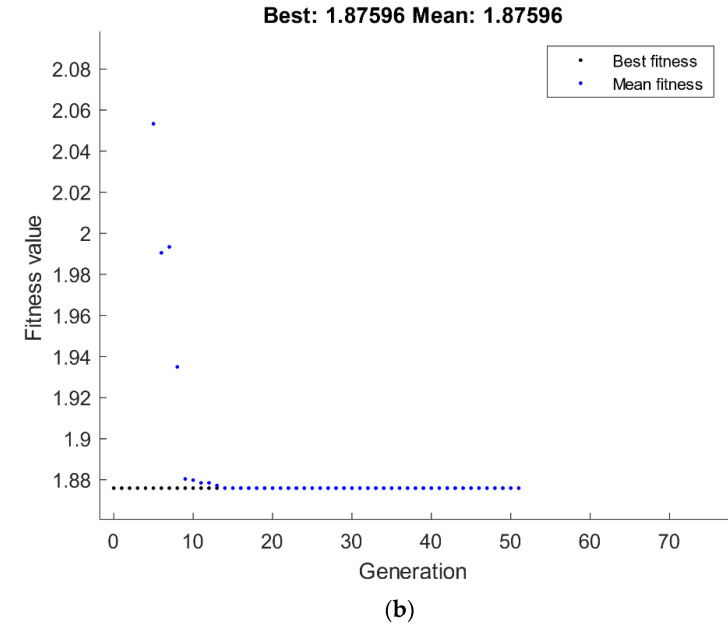
Optimum grain size by GA (**a**) and hybrid RSM-GA (**b**).

The optimization technique of GA was done through MATLAB, where the performance of fitness value and the results of the run solver view displayed the minimum possible grain size subjected to the ECAP boundary conditions. The best value of grain size by GA is 1.8759 µm, which was attained at route Bc with four passes and 120° ECAP die angle, as shown in Figure 12a. The grain size value of RSM compared with the GA technique is 1.882 µm and 1.875 µm, respectively. 

A hybrid RSM-GA was performed to enhance the obtained GA results of grain size response. The starting population of hybrid RSM-GA was based on RSM optimum ECAP conditions of four passes, 120° ECAP die angle, 0 for the dummy variable x_1_, and 1 for the dummy variable x_2_. The minimum optimum grain size value obtained from the hybrid RSM-GA is 1.875 µm, which was better than its counterpart response obtained by RSM at route Bc with four passes and 120° ECAP die angle, as shown in Figure 12b.

#### 4.2.2. Optimization of Corrosion Response

**Figure 13:**
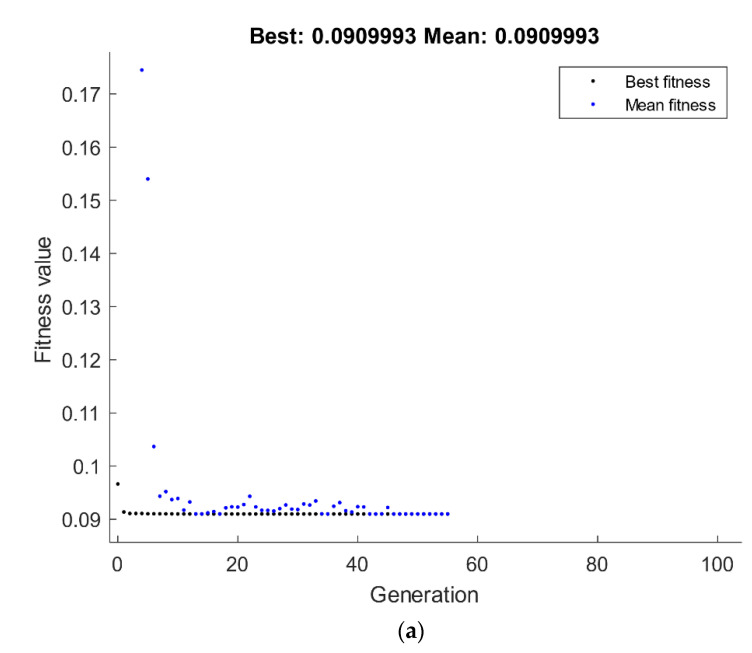

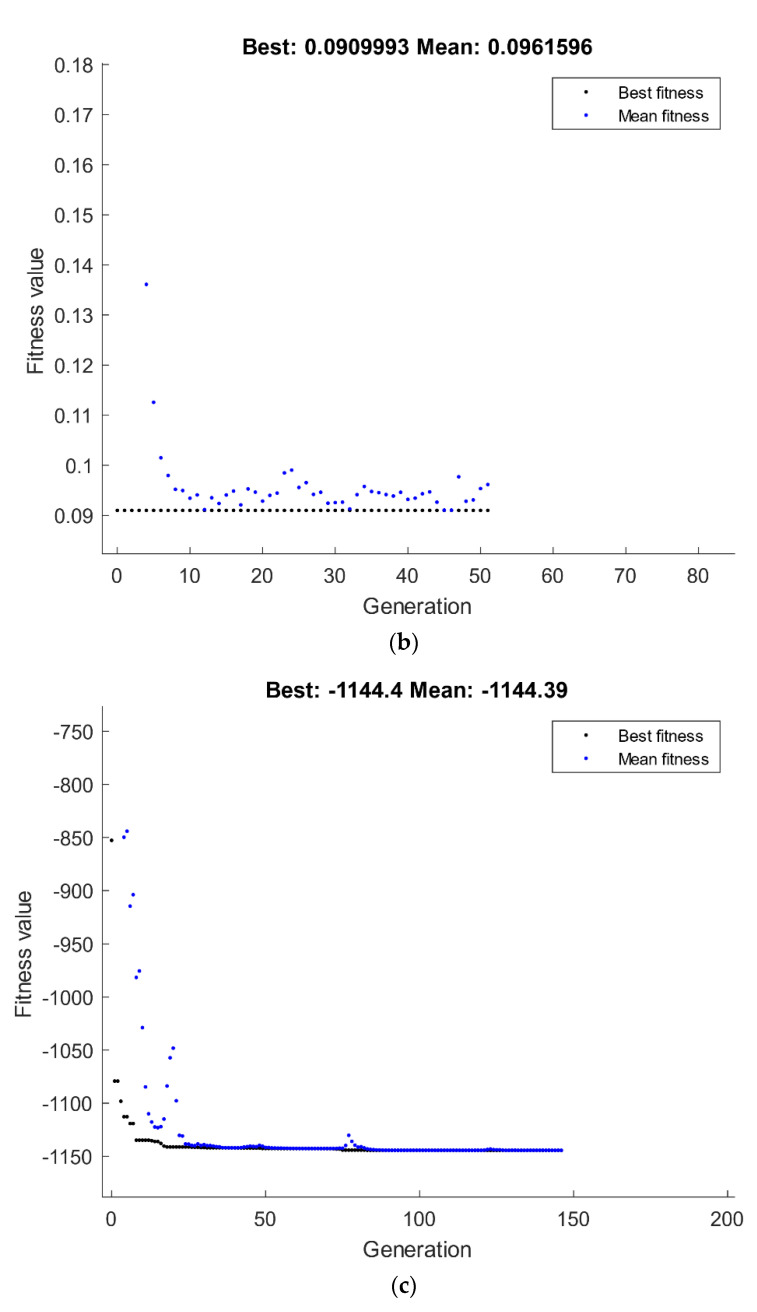

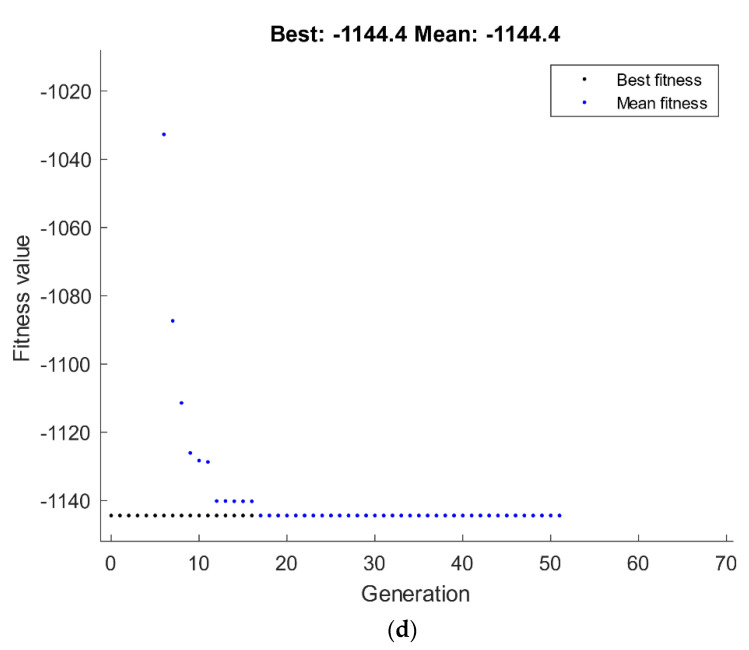
Optimum corrosion rate (**a**,**b**) and corrosion resistance (**c**,**d**) by GA (**a**,**c**) and hybrid RSM-GA (**b**,**d**).

The optimization of corrosion response by GA is shown in Figure 13. A minimization was considered for the corrosion rate response presented in Equation (5), which was set to be the fitness function and subjected to the ECAP boundary conditions of number of passes, ECAP die angle, dummy variable x_1_, and dummy variable x_2_. The best value of corrosion rate by GA is 0.0909 mpy, which was attained at route Bc with four passes and 90° ECAP die angle, as shown in Figure 13a. The corrosion rate values of RSM compared with the GA technique are 0.091 mpy and 0.090 mpy, respectively. 

Conversely, a maximization was considered for the corrosion resistance response presented in Equation (6), which was set to be the fitness function and subjected to the ECAP boundary conditions of number of passes, ECAP die angle, dummy variable x_1_, and dummy variable x_2_. The best value of corrosion resistance by GA is 1144 Ω·cm^2^, which was attained at route Bc with one pass and 120° ECAP die angle, as shown in Figure 13c. The corrosion resistance value of RSM compared with the GA technique is 1149 Ω·cm^2^ and 1144 Ω·cm^2^, respectively.

A hybrid RSM-GA was performed to enhance the obtained GA results of corrosion response. The starting population of hybrid RSM-GA was based on RSM optimum ECAP conditions of corrosion rate and resistance. The minimum optimum corrosion rate obtained from the hybrid RSM-GA (Figure 13b) is 0.090 mpy, which is better than its counterpart response obtained by RSM at route Bc with four passes and 90° ECAP die angle. Moreover, the maximum optimum corrosion resistance obtained from the hybrid RSM-GA is 1144 Ω·cm^2^ which is better than its counterpart response obtained by RSM at route Bc with one pass and 120° ECAP die angle, as shown in Figure 13d.

#### 4.2.3. Optimization of Hardness Response

**Figure 14:**
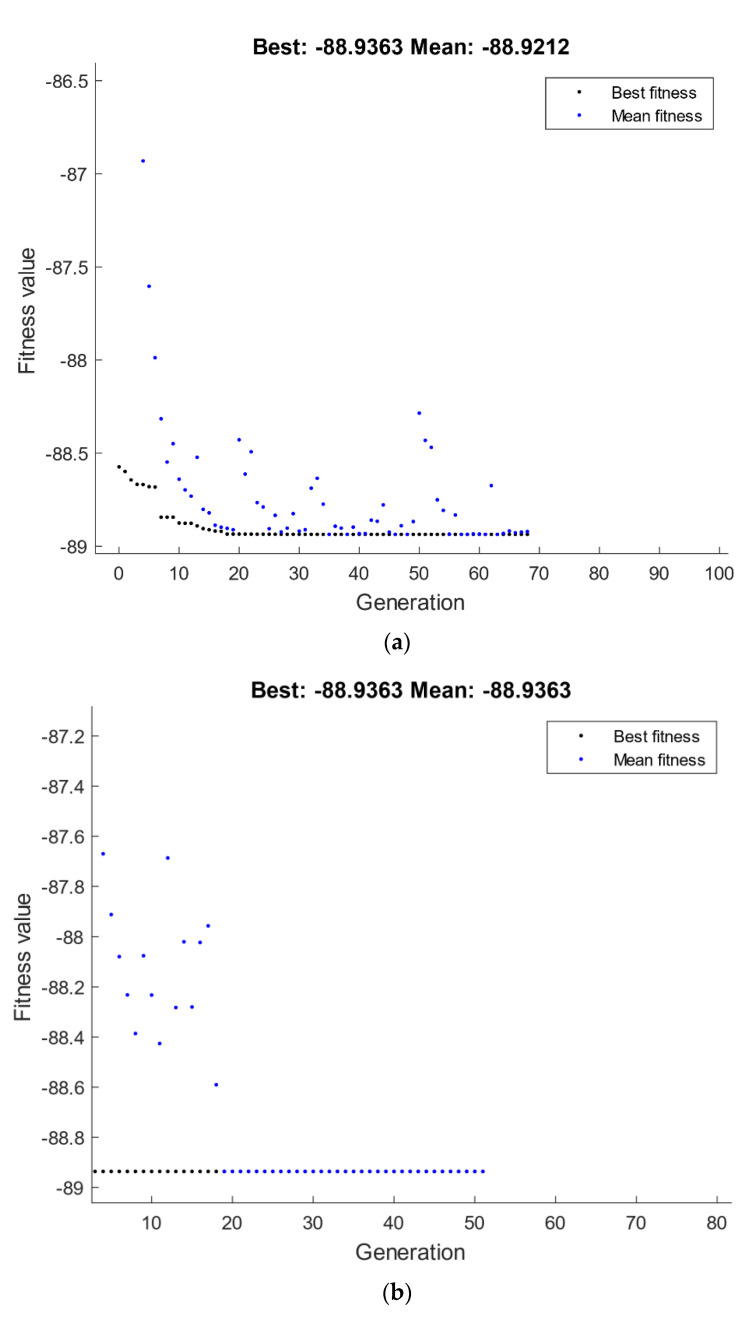

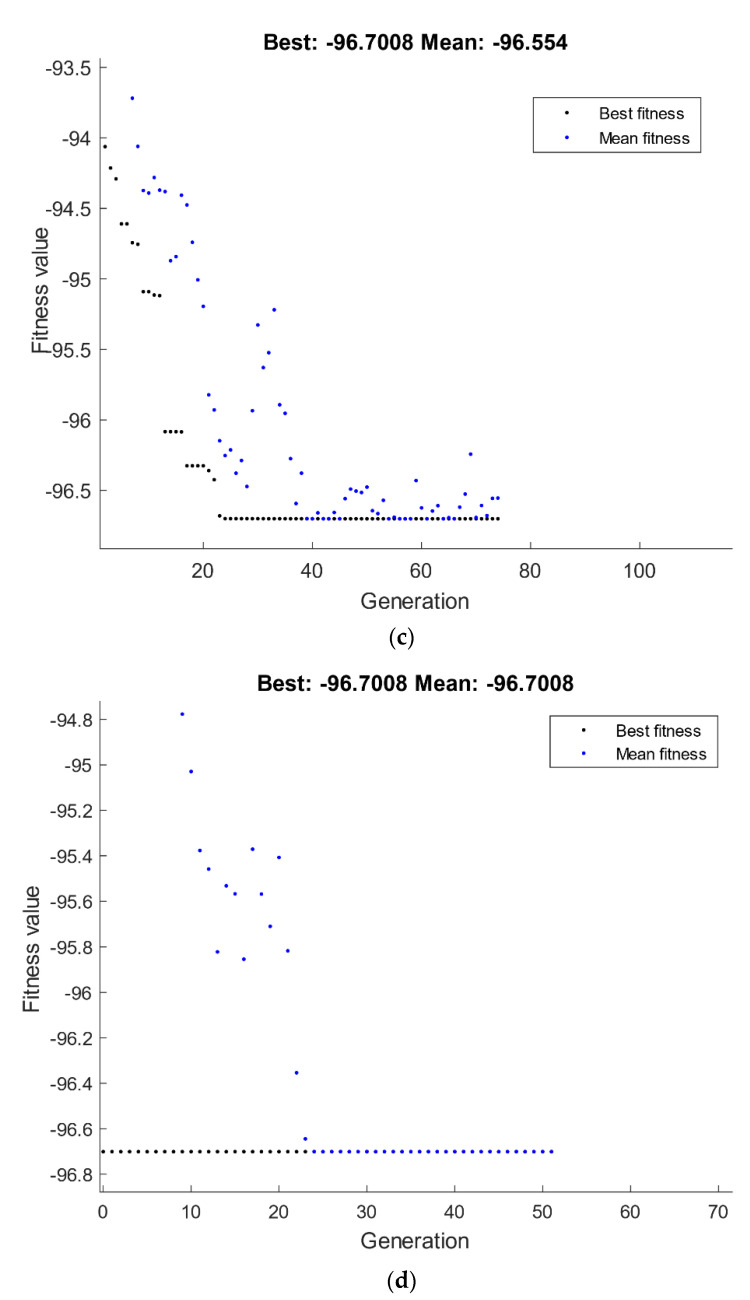
Optimum hardness at center (**a**,**b**) and edge (**c**,**d**) by GA (**a**,**c**) and hybrid RSM-GA (**b**,**d**).

The optimization of hardness response by GA is shown in Figure 14. A maximization was considered for the hardness at the center and edge responses presented in Equations (7) and (8), which were set to be the fitness functions and subjected to the ECAP boundary conditions of number of passes, ECAP die angle, dummy variable x_1_, and dummy variable x_2_. The best values of hardness at the center and edge by GA are 88.936 HV and 96.7 HV, respectively, which were attained at route Bc with four passes and 120° ECAP die angle, as shown in Figure 14a,c. 

A hybrid RSM-GA was performed to enhance the obtained GA results of hardness at the center and edge responses. The maximum optimum hardness at the center and edge values obtained from the hybrid RSM-GA are 88.936 HV and 96.7 HV, respectively, which were better than its counterpart responses obtained by RSM at route Bc with four passes and 120° ECAP die angle, as shown in Figure 14b,d.

#### 4.2.4. Optimization of Tensile Response

**Figure 15:**
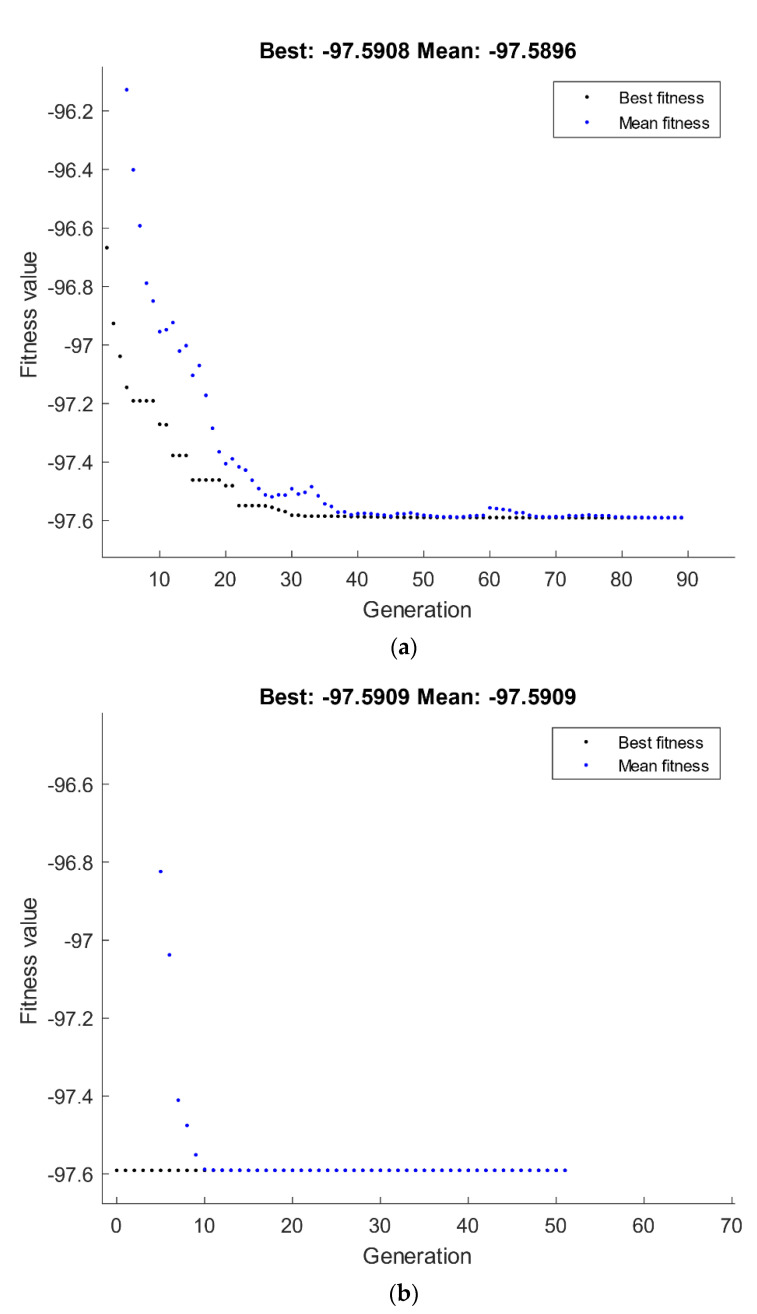

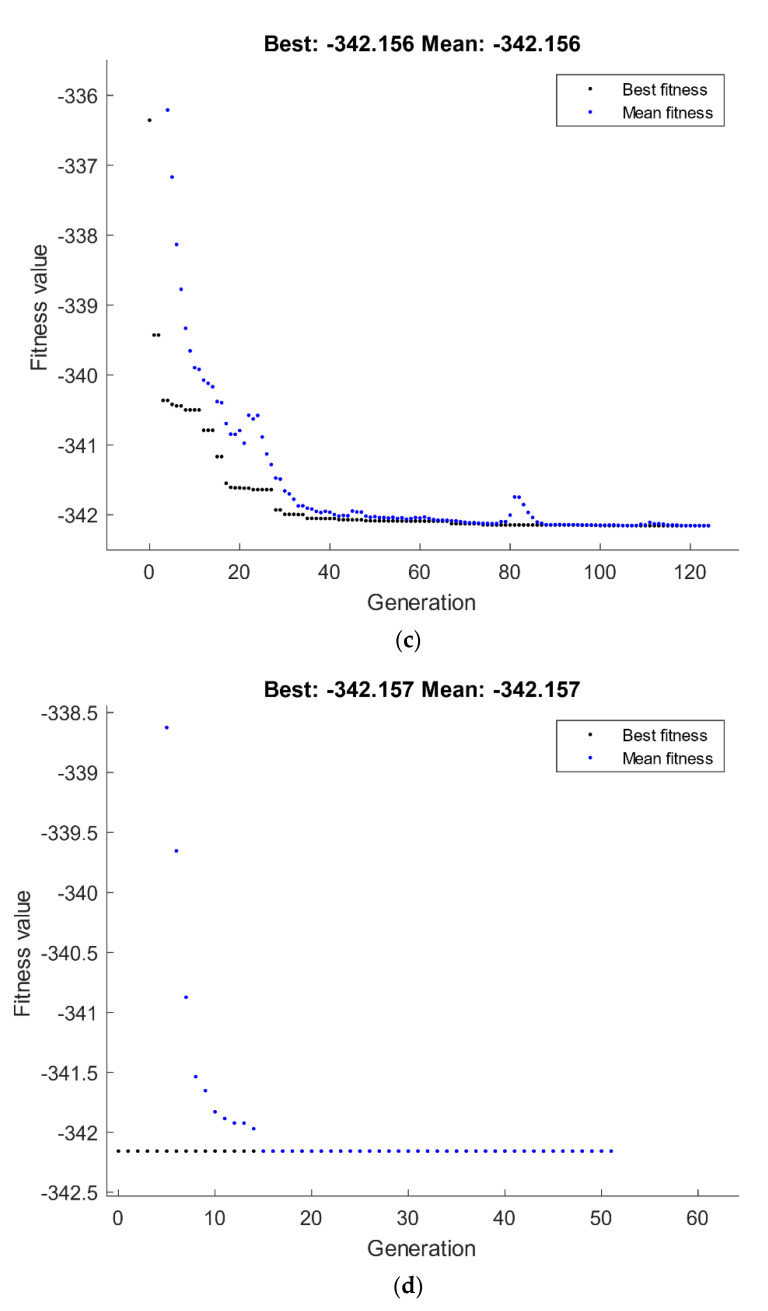

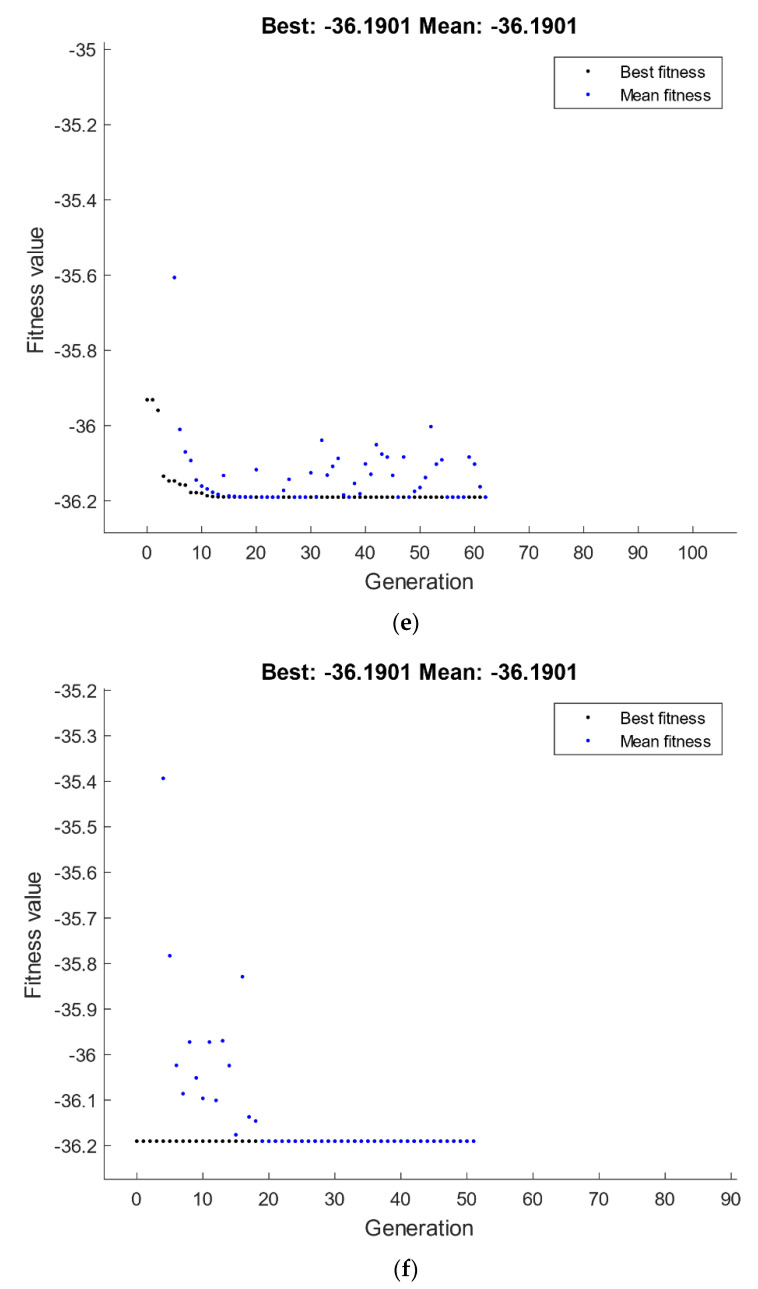
Optimum tensile response YS (**a**,**b**), TS (**c**,**d**), and D% (**e**,**f**) by GA (**a**,**c**,**e**) and hybrid RSM-GA (**b**,**d**,**f**).

The optimization of tensile response by GA is shown in Figure 15. A maximization was considered for the YS and TS responses presented in Equations (9) and (10), which were set to be the fitness functions and subjected to the ECAP boundary conditions of number of passes, ECAP die angle, dummy variable x_1_, and dummy variable x_2_. The best values of YS and TS by GA are 97.58 MPa, and 342.157 MPa, respectively, which were attained at route Bc with four passes and 90° ECAP die angle, as shown in Figure 15a,c. 

In addition, a maximization was considered for the D% percentage response presented in Equation (11), which was set to be the fitness function and subjected to the ECAP boundary conditions of number of passes, ECAP die angle, dummy variable x1, and dummy variable x2. The best value of D% by GA is 36.19, which was attained at route Bc with one pass and 120° ECAP die angle, as shown in Figure 15e.

A hybrid RSM-GA was performed to enhance the obtained GA results of YS and TS and D% percentage responses. The maximum optimum YS and TS values obtained from the hybrid RSM-GA are 97.59 MPa and 342.157 MPa, respectively, which were better than their counterparts obtained by RSM at route Bc with four passes and 90° ECAP die angle, as shown in Figure 15b,d. Moreover, the maximum optimum D% obtained from the hybrid RSM-GA is 36.19, which is better than its counterpart response obtained by RSM at route Bc with one pass and 120° ECAP die angle, as shown in Figure 15f. Table 4 summarizes the comparison of ECAP response values at experimental, RSM, GA, and hybrid RSM-GA cases.

### 4.3. Validation of GA

In this part, the optimal ECAP parameters of the various responses presented in this context of the grain size, corrosion response, hardness properties, and tensile response are displayed. The presented optimal ECAP parameters of the ECAP die angle, number of passes, and processing route types were chosen based on earlier studies in the literature of Mg-alloys that suggested the adoption of the ECAP die angle from 70° to 135° and a number of passes from one to twelve [75,77,78,79]. Table 5 shows the optimal conditions of the ECAP process for the various responses by GA and hybrid RSM-GA.

## 5. Conclusions

In this study, biodegradable Mg-Zn-Zr alloy billets were processed using ECAP utilizing two ECAP dies with internal angles of 90° and 120°. At a temperature of 250 °C, several ECAP routes (A, Bc, and C) were employed, as well as varied passes (one pass, two passes, and four passes). The influence of ECAP conditions on microstructural development, corrosion behavior, tensile characteristics, and Vicker’s microhardness was thoroughly examined. To optimize the ECAP processing parameters of an Mg-Zn-Zr alloy, RSM, ANOVA, GA, and RSM-GA were used. The following conclusions could be drawn:

1. The predicted results were very close to the actual experimental results with a narrow slight deviation.
2. The obtained regression models are adequate and could be useful to predict the optimization of ECAP parameters.
3. Route Bc is the most effective route in grain refinement
4. ECAP processing through four passes of route Bc displayed a more homogenous distribution of the ultrafine grains
5. For the multiple passes, the ECAP die angle has an insignificant effect on refining the grain size compared to the effect of the ECAP route type.
6. ECAP processing via 4Bc through the 90°-degree die revealed a better corrosion rate at 0.091mpy.
7. The 120°-die revealed higher corrosion resistance compared to the 90°-die.
8. 4Bc through the 120°-die resulted in enhancing the hardness by 86.5% relative to the AA counterpart.
9. 4Bc through the 90°-die revealed the best TS, while 2C through the 120°-die showed the best ductility at fracture.

## Figures and Tables

**Table 1:** ECAP parameters and corresponding levels.

ECAP Parameters	Parameters Levels		
	−1	0	1
Number of passes	1	2	4
ECAP die angle	90	120	
Processing route type	A	Bc	C

**Table 2:** Design of experiment of ECAP parameters.

Run	A: No. of Passes	B: Die Angle	C: Processing Route Type
1	1	120	Bc
2	2	120	A
3	4	90	C
4	2	120	C
5	2	90	Bc
6	2	120	A
7	2	90	Bc
8	4	120	Bc
9	4	120	C
10	2	120	Bc
11	1	120	C
12	4	90	Bc
13	1	90	A
14	4	90	A
15	4	90	A
16	1	90	C

**Table 3:** Grain size data of the AA and ECAPed Mg-Zn-Zr billets. All units are in µm.

	AA	90°-Die				120°-Die	
		1P	4A	4Bc	4C	1P	4Bc
Min	3.39	1.13	0.23	0.23	0.28	2.24	0.76
Max	76.73	38.10	14.53	11.76	12.73	35.22	17.86
Average	26.69	3.24	2.89	1.94	2.25	5.43	1.92
St. Deviation	14.74	2.42	1.92	1.54	1.60	4.22	1.09

**Table 4:** Summary of the results of the ECAP process.

Response		Experimental	RSM	GA	RSM-GA
Grain Size (µm)	Value	1.92	1.8821	1.8759	1.8759
	Cond.	4 passes, 120°, Route Bc	4 passes, 117°, Route Bc	4 passes, 120°, Route Bc	4 passes, 120°, Route Bc
Corrosion rate (mpy)	Value	0.091	0.09109	0.0909	0.0909
	Cond.	4 passes, 90°, Route Bc	4 passes, 90°, Route Bc	4 passes, 90°, Route Bc	4 passes, 90°, Route Bc
Corrosion resistance (Ω·cm^2^)	Value	1232	1149	1144	1144
	Cond.	1 pass, 120°, Route Bc	1 pass, 120°, Route Bc	1 pass, 120°, Route Bc	1 pass, 120°, Route Bc
Hardness at center (HV)	Value	90	88.9517	88.936	88.936
	Cond.	4 passes, 120°, Route Bc	4 passes, 120°, Route Bc	4 passes, 120°, Route Bc	4 passes, 120°, Route Bc
Hardness at edge (HV)	Value	97	96.7099	96.7008	96.7008
	Cond.	4 passes, 120°, Route Bc	4 passes, 120°, Route Bc	4 passes, 120°, Route Bc	4 passes, 120°, Route Bc
YS (MPa)	Value	98	98.0049	97.5896	97.5909
	Cond.	4 passes, 120°, Route Bc	1 pass, 90°, Route Bc	4 pass, 90°, Route Bc	4 pass, 90°, Route Bc
TS (MPa)	Value	342.4	342.156	342.157	342.157
	Cond.	4 passes, 90°, Route Bc	4 passes, 90°, Route Bc	4 passes, 90°, Route Bc	4 passes, 90°, Route Bc
D% (mm/mm)	Value	37.3	36.19	36.19	36.19
	Cond.	1 pass, 120°, Route C	1 pass, 120°, Route Bc	1 pass, 120°, Route Bc	1 pass, 120°, Route Bc

**Table 5:** Validated ECAP response based on previous studies.

Response		GA	RSM-GA
Grain Size (µm)	Value	0.6139	0.6139
	Cond.	12 passes, 135°, Route Bc	12 passes, 135°, Route Bc
Corrosion rate (mpy)	Value	0.0069	0.0069
	Cond.	12 passes, 70°, Route Bc	12 passes, 70°, Route Bc
Corrosion resistance (Ω·cm^2^)	Value	21,019.5	21,019.5
	Cond.	12 pass, 70°, Route Bc	12 pass, 70°, Route Bc
Hardness at center (HV)	Value	89.0707	89.0707
	Cond.	4 passes, 135°, Route Bc	4 passes, 135°, Route Bc
Hardness at edge (HV)	Value	178.73	178.73
	Cond.	12 passes, 135°, Route Bc	12 passes, 135°, Route Bc
YS (MPa)	Value	213.51	213.509
	Cond.	12 passes, 135°, Route Bc	12 passes, 135°, Route Bc
TS (MPa)	Value	472.153	472.153
	Cond.	12 passes, 135°, Route Bc	12 passes, 135°, Route Bc
D% (mm/mm)	Value	37.42	37.42
	Cond.	1 pass, 135°, Route Bc	1 pass, 135°, Route Bc

## Data Availability

All the raw data supporting the conclusion of this paper were provided by the authors.

## Nomenclature

ECAP	Equal channel angular pressing
RSM	Response Surface Methodology
ANOVA	Analysis of Variance
HCP	Hexagonal close-packed
SPD	Severe plastic deformation
UFG	Ultra-Fine Grain
$\varepsilon_{eq}$	The equivalent strain
ϕ	ECAP die angle
Ψ	Outer corner angle
N	Number of passes
EBSD	Electron backscatter Diffraction
(SCE)	Saturated calomel electrode
(E_corr_)	Open-circuit potential
(EIS)	Electrochemical impedance spectroscopy
Hv	Vicker’s microhardness test
GA	Genetic Algorithm
Y	Output response
f	The ECAP process independent input factors,
ε	The random error distributed about the response Y
YS	Ultimate tensile strength
TS	Tensile Strength
D	Ductility
x_1_ and x_2_	Dummy variables
R^2^	Regression Coefficient

## Appendix A

**Table A1:** Analysis of variance of grain size.

Source	DF	Grain Size (µm)			
		Sum of Squares	Mean Square	F-Value	*p*-Value
Model	7	0.1491	0.0213	78.74	<0.0001 ^a^
A-No. of Passes	1	0.0511	0.0511	188.98	<0.0001 ^a^
B-Die Angle	1	0.0029	0.0029	10.89	0.0109 ^a^
C-Dummy x_1_	1	0.0029	0.0029	10.55	0.0117 ^a^
D-Dummy x_2_	1	0.0008	0.0008	3.13	0.1147
AB	1	0.0063	0.0063	23.17	0.0013 ^a^
AC	1	0.0057	0.0057	21.18	0.0018 ^a^
AD	1	0.0025	0.0025	9.41	0.0154 ^a^
Residual	8	0.0022	0.0003		
Lack of Fit	5	0.0021	0.0004	25.92	0.0113 ^a^
Pure Error	3	0.0000	0.0000		
Cor Total	15	0.1513			
Fit Statistics		Std. Dev.	C.V. %	Adeq Precision	
		0.0164	4.76	29.8545	
		R^2^	Adjusted R^2^	Predicted R^2^	
		0.9857	0.9732	0.9155	

^a^ Within a 95% confidence interval, parameters referring to cells are significant.

**Table A2:** Analysis of variance of corrosion rate and corrosion resistance.

Source	DF	Corrosion Rate (mpy)				Corrosion Resistance (Ω·cm^2^)				
		Sum of Squares	Mean Square	F-Value	*p*-Value	DF	Sum of Squares	Mean Square	F-Value	*p*-Value
Model	9	86.58	9.62	73.35	<0.0001 ^a^	9	1.156 × 10^6^	1.284 × 10^5^	11.59	0.0037 ^a^
A-No. of Passes	1	15.46	15.46	117.88	<0.0001	1	1.674 × 10^5^	1.674 × 10^5^	15.11	0.0081
B-Die Angle	1	0.0058	0.0058	0.0441	0.8405	1	88,102.62	88,102.62	7.95	0.0304
C-Dummy x1	1	0.0425	0.0425	0.3240	0.5898	1	1217.40	1217.40	0.1099	0.7516
D-Dummy x2	1	2.50	2.50	19.08	0.0047	1	5343.05	5343.05	0.4822	0.5134
AB	1	5.07	5.07	38.63	0.0008	1	1.212 × 10^5^	1.212 × 10^5^	10.94	0.0163
AD	1	26.95	26.95	205.47	<0.0001	1	17,219.21	17,219.21	1.55	0.2590
BD	1	0.0076	0.0076	0.0578	0.8180	1	12,575.49	12,575.49	1.13	0.3277
A^2^	1	5.31	5.31	40.45	0.0007	1	2.714 × 10^5^	2.714 × 10^5^	24.49	0.0026
ABD	1	15.34	15.34	116.93	<0.0001	1	22,745.80	22,745.80	2.05	0.2019
Residual	6	0.7869	0.1311			6	66,483.81	11,080.64		
Lack of Fit	3	0.3265	0.1088	0.7093	0.6078 ^an^	3	63,550.87	21,183.62	21.67	0.0155 ^a^
Pure Error	3	0.4604	0.1535			3	2932.94	977.65		
Cor Total	15	87.36				15	1.222 × 10^6^			
Fit Statistics		Std. Dev.	C.V. %	Adeq Precision			Std. Dev.	C.V. %	Adeq Precision	
		0.3621	9.87	34.1733			105.26	17.45	11.8548	
		R^2^	Adjusted R^2^	Predicted R^2^			R^2^	Adjusted R^2^	Predicted R^2^	
		0.9910	0.9775	0.9846			0.9456	0.8640	0.8798	

^a^ Within a 95% confidence interval, parameters referring to filled cells are the significant, ^an^ are the insignificant terms.

**Table A3:** Analysis of variance of hardness at the center and edge.

Source	DF	Hardness at Center (HV)				Source	DF	Hardness at Edge (HV)			
		Sum of Squares	Mean Square	F-Value	*p*-Value			Sum of Squares	Mean Square	F-Value	*p*-Value
Model	6	0.0004	0.0001	95.70	<0.0001 ^a^	Model	7	0.0002	0.0000	63.99	<0.0001 ^a^
A-No. of Passes	1	0.0002	0.0002	372.73	<0.0001	A-No. of Passes	1	0.0001	0.0001	198.13	<0.0001
B-Die Angle	1	0.0000	0.0000	46.84	<0.0001	B-Die Angle	1	0.0000	0.0000	72.47	<0.0001
C-Dummy X1	1	0.0000	0.0000	50.78	<0.0001	C-Dummy X1	1	9.004 × 10^−6^	9.004 × 10^−6^	25.34	0.0010
D-Dummy X2	1	3.150 × 10^−6^	3.150 × 10^−6^	5.03	0.0516	D-Dummy X2	1	5.651 × 10^−6^	5.651 × 10^−6^	15.91	0.0040
BC	1	0.0000	0.0000	49.59	<0.0001	AB	1	5.436 × 10^−6^	5.436 × 10^−6^	15.30	0.0045
A^2^	1	0.0000	0.0000	19.34	0.0017	AD	1	2.090 × 10^−6^	2.090 × 10^−6^	5.88	0.0415
Residual	9	5.637 × 10^−6^	6.263 × 10^−7^			BC	1	0.0000	0.0000	64.43	<0.0001
Lack of Fit	6	5.480 × 10^−6^	9.133 × 10^−7^	17.42	0.0197 ^a^	Residual	8	2.842 × 10^−6^	3.553 × 10^−7^		
Pure Error	3	1.573 × 10^−7^	5.243 × 10^−8^			Lack of Fit	5	1.501 × 10^−6^	3.001 × 10^−7^	0.6711	0.6756 ^an^
Cor Total	15	0.0004				Pure Error	3	1.342 × 10^−6^	4.472 × 10^−7^		
						Cor Total	15	0.0002			
Fit Statistics		Std. Dev.	C.V. %	Adeq Precision		Fit Statistics		Std. Dev.	C.V. %	Adeq Precision	
		0.0008	0.7072	24.5495				0.0006	0.5560	26.6897	
		R^2^	Adjusted R^2^	Predicted R^2^				R^2^	Adjusted R^2^	Predicted R^2^	
		0.9846	0.9743	0.9481				0.9825	0.9671	0.9245	

^a^ Within a 95% confidence interval, parameters referring to filled cells are significant, ^an^ are insignificant terms.

**Table A4:** Analysis of variance of YS and TS of ZK30.

Source	DF	YS (MPa)				Source	DF	TS (MPa)			
		Sum of Squares	Mean Square	F-Value	*p*-Value			Sum of Squares	Mean Square	F-Value	*p*-Value
Model	6	312.95	52.16	20.60	<0.0001 ^a^	Model	6	2931.98	488.66	48.53	<0.0001 ^a^
A-No. of Passes	1	35.12	35.12	13.87	0.0047	A-No. of Passes	1	873.21	873.21	86.72	<0.0001
B-Die Angle	1	80.39	80.39	31.75	0.0003	B-Die Angle	1	953.57	953.57	94.70	<0.0001
C-Dummy x_1_	1	4.06	4.06	1.60	0.2374	C-Dummy x_1_	1	206.71	206.71	20.53	0.0014
D-Dummy x_2_	1	38.89	38.89	15.36	0.0035	D-Dummy x_2_	1	9.10	9.10	0.9035	0.3667
AB	1	46.53	46.53	18.38	0.0020	AB	1	384.40	384.40	38.18	0.0002
A^2^	1	10.25	10.25	4.05	0.0751	AD	1	34.03	34.03	3.38	0.0992
Residual	9	22.79	2.53			Residual	9	90.62	10.07		
Lack of Fit	6	20.02	3.34	3.62	0.1591 ^an^	Lack of Fit	6	69.12	11.52	1.61	0.3738 ^an^
Pure Error	3	2.77	0.9217			Pure Error	3	21.50	7.17		
Cor Total	15	335.74				Cor Total	15	3022.60			
Fit Statistics		Std. Dev.	C.V. %	Adeq Precision		Fit Statistics		Std. Dev.	C.V. %	Adeq Precision	
		1.59	1.74	12.5037				3.17	0.9804	19.0151	
		R^2^	Adjusted R^2^	Predicted R^2^				R^2^	Adjusted R^2^	Predicted R^2^	
		0.9321	0.8869	0.7426				0.97	0.95	0.9067	

^a^ Within a 95% confidence interval, parameters referring to filled cells are significant, ^an^ are insignificant term.

**Table A5:** Analysis of variance of D% percentage of ZK30.

Source	DF	D% (mm/mm)			
		Sum of Squares	Mean Square	F-Value	*p*-Value
Model	7	145.30	20.76	74.74	<0.0001 ^a^
A-No. of Passes	1	13.56	13.56	48.84	0.0001
B-Die Angle	1	21.04	21.04	75.76	<0.0001
C-Dummy x_1_	1	1.01	1.01	3.62	0.0934
D-Dummy x_2_	1	20.52	20.52	73.88	<0.0001
AC	1	8.76	8.76	31.55	0.0005
AD	1	9.17	9.17	33.03	0.0004
A^2^	1	4.37	4.37	15.73	0.0041
Residual	8	2.22	0.2777		
Lack of Fit	5	1.64	0.3273	1.68	0.3554 ^an^
Pure Error	3	0.5850	0.1950		
Cor Total	15	147.52			
Fit Statistics		Std. Dev.	C.V. %	Adeq Precision	
		0.5270	1.57	29.4038	
		R^2^	Adjusted R^2^	Predicted R^2^	
		0.9849	0.9718	0.9410	

^a^ Within a 95% confidence interval, parameters referring to filled cells are significant, ^an^ are insignificant terms.
